# Evolution of a General RNA-Cleaving FANA Enzyme

**DOI:** 10.1038/s41467-018-07611-1

**Published:** 2018-11-29

**Authors:** Yajun Wang, Arlene K. Ngor, Ali Nikoomanzar, John C. Chaput

**Affiliations:** 1 Department of Pharmaceutical Sciences, University of California, Irvine, CA 92697-3958 USA; 2 Department of Chemistry, University of California, Irvine, CA 92697-3958 USA; 3 Department of Molecular Biology and Biochemistry, University of California, Irvine, CA 92697-3958 USA

## Abstract

The isolation of synthetic genetic polymers (XNAs) with catalytic activity demonstrates that catalysis is not limited to natural biopolymers, but it remains unknown whether such systems can achieve robust catalysis with Michaelis-Menten kinetics. Here, we describe an efficient RNA-cleaving 2’-fluoroarabino nucleic acid enzyme (FANAzyme) that functions with a rate enhancement of >10^6^-fold over the uncatalyzed reaction and exhibits substrate saturation kinetics typical of most natural enzymes. The FANAzyme was generated by in vitro evolution using natural polymerases that were found to recognize FANA substrates with high fidelity. The enzyme comprises a small 25 nucleotide catalytic domain flanked by substrate-binding arms that can be engineered to recognize diverse RNA targets. Substrate cleavage occurs at a specific phosphodiester bond located between an unpaired guanine and a paired uracil in the substrate recognition arm. Our results expand the chemical space of nucleic acid enzymes to include nuclease-resistant scaffolds with strong catalytic activity.

## Abstract (web summary)

Artificial genetic polymers (XNAs) have been explored for their nuclease activity, but XNAzymes have proven challenging to discover by in vitro selection. Here, the authors generate an efficient RNA-cleaving 2’-fluoroarabino nucleic acid enzyme (FANAzyme) by in vitro evolution using natural DNA polymerases.

## Introduction

Nucleic acid enzymes are highly proficient catalysts at promoting sequence-specific RNA cleavage. In addition to the well-known catalytic motifs found in natural RNAs (e.g., group I intron, hammerhead, and hairpin)^1^, in vitro selection techniques have produced examples of both RNA and DNA enzymes (ribozymes and deoxyribozymes) with strong ribonuclease activity^2–4^. One of these enzymes, commonly referred to as the 10–23 DNA enzyme, can be made to function as a general purpose RNA-cleaving DNA enzyme^4,5^. Although 10–23 has been used to silence the expression of numerous pathological RNAs^6^, including targets associated with T helper type 2-driven asthma and basal-cell carcinoma^7,8^, the in vitro application of this and other related enzymes are ultimately limited by the intrinsic biological stability of DNA and RNA, which are prone to nuclease digestion. While this problem can be overcome with chemical modifications that are introduced post-selection^9^, care must be taken not to disrupt the activity of the catalytic domain.

Artificial genetic polymers, commonly referred to as xeno-nucleic acids (XNAs), provide an alternative solution to the problem of biological stability by yielding nucleic acid molecules with backbone structures that are resistant or, in some cases, recalcitrant to nuclease digestion^10^. In recent years, several XNA aptamers have been isolated by Darwinian evolution methods that use engineered polymerases to convert genetic information back and forth between DNA and XNA^11,12^. In a particularly striking example, an aptamer produced by this process was shown to function in the presence of a strong nucleolytic enzyme, demonstrating a critical aspect of biostability^13^. Similar successes have also been achieved using mirror-image aptamers (i.e., spiegelmers)^14^, but such reagents are restricted to achiral targets or targets with mirror-images that can be generated by chemical synthesis^15^. Since XNA aptamers are not restricted in this way, they can be used to target a broader range of biological molecules, most notably large proteins and cells that cannot be produced by chemical synthesis^16^.

Despite the growing success of XNA aptamers, XNA enzymes (XNAzymes) have proven substantially more challenging to discover by in vitro selection. All of the XNA enzymes generated thus far, which includes catalysts generated from four different backbone chemistries (arabino nucleic acid (ANA), 2’-fluoroarabino nucleic acid (FANA), hexitol nucleic acid (HNA), and cyclohexenyl nucleic acid (CeNA)) and two different enzymatic activities (RNA ligation and cleavage), function with catalytic rates that are slower than their equivalent DNA and RNA enzymes^17^. Although one enzyme (a FANA ribonuclease) exhibited a modest rate of 0.02 min^−1^ in buffer containing 50 mM MgCl_2_ (pH 8.5), most of the other examples function with rates that are closer to 0.0001 min^−1^. Moreover, none of the existing XNA enzymes have been shown to function with Michaelis–Menten kinetics, which suggests that their substrate binding affinity (*K*_M_) maybe unsuitable for saturation kinetics across a range of substrate concentrations. This observation raises the question of whether XNAs are inherently limited in their ability to fold into complex tertiary structures capable of achieving robust catalytic activity or, alternatively, whether XNA enzymes are simply constrained by the enzymes used to replicate them under in vitro selection conditions. The former of these may have contributed to nature’s selection of ribofuranosyl nucleic acids as the molecular basis of life’s genetic system^18^, while the latter is a mechanical problem that can be improved through the continued development of better XNA polymerases.

**Fig. 1:**
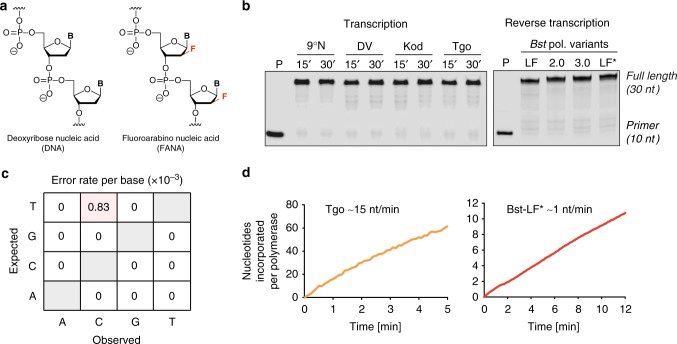
FANA transcription and reverse transcription in vitro. **a** Constitutional structures for 2’-deoxyribonucleic acid (DNA) and 2’-fluoroarabino nucleic acid (FANA). **b** FANA transcription activity for wild-type archaeal DNA polymerases (exo−) from 9°N, DV, Kod, and Tgo (left panel). Samples were analyzed after 15 and 30 min at 55 °C. FANA reverse transcriptase activity of Bst DNA polymerase LF, 2.0, 3.0, and LF* (right panel). LF* denotes wild-type Bst DNA polymerase, large fragment, expressed and purified from *E. coli*. Samples were analyzed after 30 min at 50 °C. All samples were resolved on denaturing PAGE and visualized using a LI-COR Odyssey CLx. **c** Fidelity profile observed for FANA replication using Tgo and Bst LF* polymerases. The mutation profile reveals a mutation rate of 8 × 10^-4^ and an overall fidelity of ~99.9%. **d** Catalytic rates observed for FANA synthesis with Tgo (left panel) and reverse transcription with Bst LF* (right panel)

Here we describe the evolution of a FANA enzyme (FANAzyme) that was isolated from an unbiased pool of ~10^14^ unique FANA sequences (Fig. 1a). The selection was performed using natural DNA polymerases that were discovered to transcribe and reverse transcribe FANA sequences with high efficiency and fidelity. The enzyme cleaves RNA at a specific phosphodiester bond with a catalytic rate that is >10^6^-fold faster than the uncatalyzed reaction and achieves substrate saturation with Michaelis–Menten kinetics. Divalent metal ion, pH profiles, and mass spectrometric (MS) analysis indicate that the reaction follows a metal and pH-dependent transesterification mechanism to produce an upstream cleavage product carrying a cyclic 2’,3’-monophosphate and a downstream strand with a 5’ OH group. The enzyme was shown to be generalizable to other RNA targets by changing the sequence of the substrate-binding domain. These data demonstrate that FANA is capable of folding into structures with robust catalysis, which provides a framework for evolving new types of XNA enzymes.

## Results

### Polymerase synthesis and reverse transcription of FANA

In vitro selection protocols require robust and faithful polymerases to synthesize and reverse transcribe diverse populations of sequences^19,20^. Recognizing that FANA is a close structural analog of DNA (Fig. 1a)^21^, we postulated that many of the critical enzyme–substrate contacts required for a DNA polymerase to recognize a DNA/DNA homoduplex would likely be maintained in a FANA/DNA heteroduplex^22^. We were particularly interested in evaluating the properties of replicative DNA polymerases isolated from hyperthermophilic species of archaea, as the exonuclease-deficient versions (exo−) of these enzymes are known to exhibit an increased tolerance for sugar-modified substrates^23–27^, including FANA^28^. The most common examples include, polymerases isolated from *Thermococcus* sp. 9°N (9°N), *Pyrococcus* sp. deep vent (DV), *Thermococcus gorgonarius* (Tgo), and *Thermococcus kodakarensis* (Kod).

To compare the efficiency of FANA synthesis, we used a primer-extension assay in which natural DNA polymerases were challenged to extend a DNA primer annealed to a DNA template with commercial 2’-fluoroarabino NTP substrates. The primer–template complex consisted of a 10 nucleotide (nt) primer-binding site followed by an unpaired region of 20 nts. The results of our screen for FANA polymerase activity are shown in Fig. 1b. Remarkably, all of the enzymes tested (9°N, DV, Tgo, and Kod) exhibited rapid full-length primer extension after a brief 15 min incubation at 55 °C in standard polymerase buffer devoid of mutagenic manganese ions, commonly used to relax the specificity of natural DNA polymerases.^29^ This result was recapitulated with a DNA library that contained a random region of 40 sequential positions flanked on both sides with fixed-sequence primer-binding sites. In all cases, the archaeal DNA polymerases were found to copy the DNA library into FANA after a 1-h incubation at 55 °C (Supplementary Fig. 1). The absence of any significant loss of activity as the enzyme progressed from the DNA primer into the FANA-extended region supports the prediction that FANA/DNA heteroduplexes are structurally similar to DNA/DNA homoduplexes.

Next, we sought to identify a DNA polymerase that could function with reverse transcriptase activity by copying FANA templates back into DNA. This step is necessary to complete the replication cycle required for in vitro selection. For this assay, we used a chimeric DNA/FANA primer–template complex of identical length and sequence as the previous DNA primer–template complex. However, the DNA template was replaced with a FANA oligonucleotide generated by solid-phase synthesis. Our analysis examined variants of a *Geobacillus stearothermophilus* (*Bst*) DNA polymerase I large fragment (LF), which has been shown to recognize a number of sugar-modified substrates^30–33^. We evaluated three commercial versions of Bst DNA polymerase (LF, 2.0, and 3.0 from New England Biolabs), along with a second version of the wild-type LF polymerase (LF*) that was expressed and purified from *Escherichia coli*^34^. Surprisingly, each of the enzymes tested exhibited strong FANA reverse transcriptase activity following a short 30 min incubation at 50 °C with dNTP substrates (Fig. 1b). Similar to the enzymes used for FANA synthesis, the results observed on a short well-defined template were extended to a random sequence library (Supplementary Fig. 1). The ability to synthesize and reverse transcribe FANA libraries with high primer-extension efficiency demonstrates that certain natural DNA polymerases are able to recognize FANA substrates with low template-sequence bias.

We measured the fidelity and rates of FANA synthesis and reverse transcription to gain further insights into how natural DNA polymerases recognize FANA substrates. Polymerase fidelity was measured by sequencing the product of a complete cycle of FANA synthesis and reverse transcription (Supplementary Fig. 2)^35^. This assay measures the aggregate fidelity of replication (DNA→FANA→DNA), which is operationally different than the more restricted view of fidelity as the accuracy of single-nucleotide incorporation. Several controls were implemented to ensure that the fidelity values represented the true fidelity of FANA synthesis, including the use of a T-T mismatch in the primer region that resulted in an T→A transversion when the FANA strand was reverse transcribed back into DNA. Alignment of the resulting sequences (Supplementary Fig. 2) reveals one T-to-C mutation (Fig. 1c) and one deletion in a sampling of 1200 nt positions, corresponding to a mutation rate of ~ 8 × 10^−4^ and an overall fidelity of 99.9%. These values compare favorably with known XNA polymerases^11^, which exhibit error rates in the range of 4.3 × 10^−3^ to 5.3 × 10^−2^.

In parallel, we measured the average rate of FANA transcription and reverse transcription using natural Tgo and Bst LF* DNA polymerases, respectively, as a representative polymerase pair. Measurements were performed in real time using polymerase kinetic profiling (PKPro), a technique that monitors nucleotide synthesis using high-resolution melting fluorescent dyes that intercalate into the growing duplex^36^. Accordingly, we found that Tgo-mediated FANA synthesis on DNA templates occurs at a rate of ~15 nt/min and Bst LF*-mediated DNA synthesis on FANA templates occurs at a slightly slower rate of ~1 nt/min (Fig. 1d). Although the rate of Tgo DNA polymerase is five-fold slower than Tgo-D4K (an engineered FANA polymerase)^36^, the natural Tgo DNA polymerase functions with superior fidelity, making it a better polymerase for FANA synthesis.

### In vitro selection of RNA-cleaving FANA enzymes

**Fig. 2:**
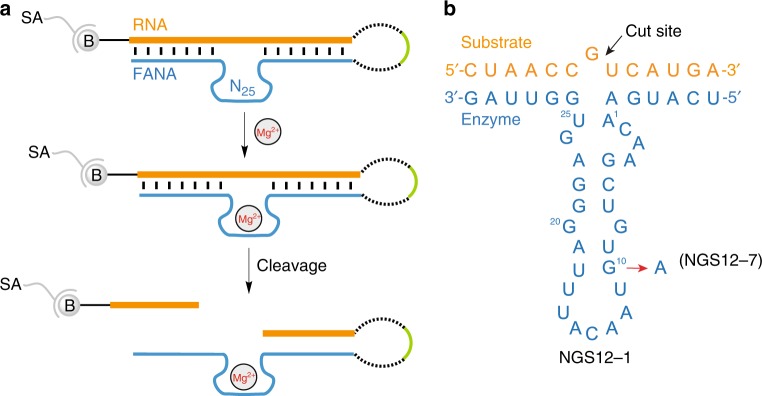
In vitro selection of an RNA-cleaving FANA enzyme. **a** In vitro selection strategy. A population of 10^14^ FANA molecules (blue) linked to an RNA substrate (orange) through a flexible DNA linker (black dashed line) that was immobilized on streptavidin (SA)-coated magnetic beads were incubated with magnesium ions to identify FANA enzymes capable of RNA cleavage. Self-cleaving FANA enzymes were subjected to iterative cycles of selection and amplification to enrich for individual catalyst with strong RNA cleaving activity. The selection progress was monitored by fluorescence using an internal FAM label (green) located in the DNA loop. **b** Predicted secondary structure of FANAzyme NGS12-1 (blue) and its mutant version NGS12-7 in complex with a 13-nt RNA substrate (orange). The cleavage site at the unpaired ribo-G is indicated by a black arrow

Iterative rounds of in vitro selection and amplification were performed starting from an unbiased library of 10^14^ different FANA molecules using a self-cleavage strategy (Fig. 2a) developed by Breaker and Joyce for the evolution of RNA-cleaving DNA enzymes^37^. Each molecule contained a 5’ biotin moiety, followed by a short DNA spacer, then a 13-nt RNA substrate linked to a 20-nt DNA loop with an internal fluorescein label, and finally 25 random FANA nucleotides flanked by a fixed-sequence region that was complementary to the RNA target (Supplementary Fig. 3). For each round of selection, the molecules were applied to a streptavidin-coated solid support as double-stranded material constructed by Tgo-mediated FANA synthesis and the DNA template was removed with cold solutions of 0.1 M NaOH and 1 mM EDTA. The beads were neutralized and functional FANA catalysts were eluted with a solution containing 20 mM MgCl_2_ at pH 7.5 and 24 °C. This approach resulted in the cleavage of a predefined G-U phosphodiester bond located in the RNA substrate and release of the 3’ cleavage product into the eluate. The small number of FANA catalysts present in the starting population were isolated, reverse transcribed back into DNA, and amplified by PCR using the fixed sequences flanking the randomized region. The amplified DNA was made single stranded and used to generate a new library of FANA molecules for input into the next round of selection.

The selective amplification procedure was repeated to promote the enrichment of RNA-cleaving FANA catalysts. Over the course of the selection, the magnesium concentration was reduced from 20 to 2 mM and the incubation time was reduced from 18 h to 30 min (Supplementary Fig. 4). To evaluate the molecules that survived the selection, we cloned and sequenced 16 individual molecules from the 12th round of selection, which revealed 11 unique motifs, one of which was present five times (Supplementary Table 2). To gain further insight into the enriched population of molecules, cDNA from the 12th round of selection was subjected to next-generation sequencing (NGS) using an Illumina HiSeq platform (Supplementary Table 3). The most abundant sequence identified by NGS analysis matched the most abundant sequence observed by Sanger sequencing, indicating that the selection protocol had likely reached an enrichment plateau.

We surveyed all 11 motifs identified by Sanger sequencing and the top 7 most abundant motifs identified by NGS analysis for RNA cleavage activity under bimolecular *in-trans* cleavage conditions. Among the tested motifs, only the most abundant sequence (NGS12-1) and its close analog bearing a single point mutation (NGS12-7) displayed RNA cleavage activity (Supplementary Tables 2 and 3). The predicted secondary structure of this FANAzyme is provided in Fig. 2b. The catalytic domain consists of a stem-loop structure that contains a G-G mismatch (G_8_–G_20_) in the Watson–Crick duplex. The predicted structural difference between NGS12-1 and NGS12-7 is the sequence change from a G**•**U wobble pair (G_10_–U_18_) to a standard A-U Watson–Crick pair (A_10_–U_18_), respectively.

### Biochemical characterization

**Fig. 3:**
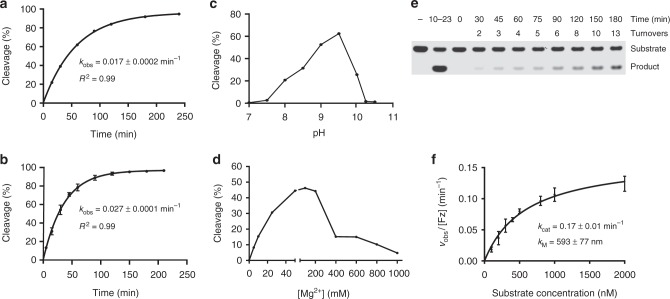
Enzymatic parameters of FANA enzymes. **a**, **b** Pre-steady state kinetic plots of NGS12-1 and NGS12-7 in buffer (pH 8.5) containing 25 mM MgCl_2_ and 200 mM NaCl at 24 °C ([Fz] = 2.5 µM, [*S*] = 0.5 µM; *n* = 3; error bars represent standard deviation, s.d.). **c**, **d** pH and Mg^2+^ activity profile of NGS12-7 at 24 °C. **e** Representative gel of NGS12-7 showing RNA cleavage activity under multiple-turnover conditions at pH 8.5, 24 °C ([Fz] = 10 nM, [*S*] = 0.5 µM). **f** Michaelis–Menten kinetic profile of NGS12-7. *n* = 3; *R*^2^ ≥0.95

To assess the biochemical properties of the selected FANAzyme, we used the same bimolecular *in-trans* construct used to evaluate individual clones to measure the pseudo first-order rate constants (*k*_obs_) for NGS12-1 and NGS12-7 under single-turnover conditions (2.5 µM FANAzyme, 0.5 µM RNA) in buffer containing 25 mM MgCl_2_ at pH 8.5 and 24 °C. Our results indicate that FANAzymes NGS12-1 and NGS12-7 function with *k*_obs_ values of 0.017 and 0.027 min^−1^, respectively (Fig. 3, Supplementary Fig. 5). The slightly faster rate (1.5-fold) of NGS12-7 is presumably due to the A-U Watson–Crick base pair, which replaces a G**•**U wobble in the stem loop of the catalytic domain. Interestingly, the DNA version of NGS12-7 was completely inactive when tested after 22 h of incubation, confirming a compositional requirement of FANA for the catalyst to function (Supplementary Fig. 6). High-resolution exact mass characterization of the upstream cleavage fragment of the RNA substrate using electrospray ionization (ESI) revealed an RNA product containing a terminal 2’,3’-cyclic monophosphate group, indicating that the reaction follows a transesterification mechanism with 2’-anchimeric assistance (Supplementary Fig. 7). Given its higher level of activity, subsequent studies were performed with NGS12-7.

Next, we determined the pH and magnesium requirements for NGS12-7. Functional assays performed across a pH range of 7–10.5 gave a bell-shaped curve with a pH optimum of 9.5 (Fig. 3c), which is consistent with a transesterification mechanism involving nucleophilic activation by deprotonation of the adjacent 2’-OH group. A screen of magnesium concentrations from 0 to 1 M demonstrates that the catalytic activity of NGS12-7 was half-maximal at ~20 mM Mg^2+^, with maximum activity achieved at 50–100 mM MgCl_2_ (Fig. 3d). Above 400 mM MgCl_2_, activity levels dropped precipitously, suggesting either aggregation or changes in the folded state of NGS12-7.

NGS12-7 is capable of cleaving RNA under multiple-turnover conditions (Fig. 3e). By performing this analysis across a range of substrate concentrations, a Michaelis–Menten plot was generated displaying the catalytic properties of NGS12-7 under standard cleavage conditions (pH 8.5, 200 mM NaCl, 25 mM MgCl_2_, 24 °C). The resulting curve shown in Fig. 3f reveals a maximum rate constant (*k*_cat_) of ~0.2 ± 0.01 min^−1^ and a *K*_M_ of ~600 ± 77 nM. The *k*_cat_ and *K*_M_ observed for NGS12-7 are similar to many natural and in vitro selected RNA-cleaving ribozymes and deoxyribozymes.^38–40^ Since the rate constant for the uncatalyzed reaction is ~10^−8^—10^−7^ min^−1^,^41,42^ we estimate that NGS12-7 achieves a rate enhancement (*k*_cat_/*k*_uncat_) of 10^6^–10^7^-fold over the uncatalyzed reaction. To our knowledge, this is the first example of a Michaelis–Menten profile obtained for an in vitro selected XNA enzyme. More importantly, the high catalytic activity of NGS12-7 (*k*_cat_ ~ 0.2 min^−1^) demonstrates that FANA is not limited in its ability to fold into shapes with high catalytic activity, which has critical implications for both basic and applied areas of research.

**Fig. 4:**
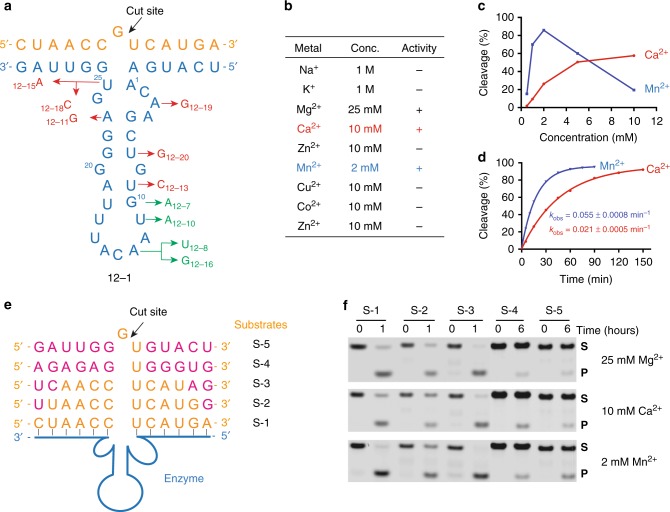
Functional analysis of FANAzymes. **a** Structure–activity profile of NGS12-1 showing functional mutations (green) and non-functional mutations (red) observed in highly abundant NGS sequences. **b** Monovalent and bivalent metal ion requirements for NGS12-7. **c** Mn^2+^ or Ca^2+^ activity profile of NGS12-7. **d** Pre-steady state kinetic plots of NGS12-7 in the presence of 2 mM MnCl_2_ or 10 mM CaCl_2_ in buffer (pH 8.5) containing 200 mM NaCl at 24 °C ([Fz] = 2.5 µM, [*S*] = 0.5 µM). **e**, **f** Substrate specificity of NGS12-7. **e** Engineered versions of NGS12-7 in which the substrate-binding arms were modified to recognize different RNA substrates. **f** Gel images showing the cleavage activity of engineered NGS12-7 variants toward different RNA substrates. Reactions were performed in buffer (pH 8.5) containing 200 mM NaCl supplemented with the labeled bivalent metal ion at 24 °C (Fz) = 2.5 µM, [*S*] = 0.5 µM). S substrate, P product

Eleven of the 20 most abundant sequences identified by NGS analysis were highly homologous to NGS12-7 (Supplementary Table 4). For these motifs, a structure–activity relationship (SAR) was established by evaluating the RNA-cleaving activity of each variant in cleavage buffer containing 25 mM MgCl_2_ at pH 8.5 and 24 °C. The SAR profile summarized in Fig. 4a indicates that residues close to the cleavage site are highly conserved, while distal positions are more permissive. This trend is clearly shown by the fact that point mutations made to positions near the cleavage site are inactive, while mutations made in the loop are functional. Interestingly, none of the mutant enzymes showed stronger activity than NGS12-7, which supports the notion that sequence abundance in NGS data can serve as an effective predictor of catalytic activity among homologous nucleic acid enzymes^43^.

We evaluated the monovalent and divalent metal ion dependence of NGS12-7 under single-turnover conditions at pH 8.5 and 24 °C (Fig. 4b). The enzyme is highly dependent on the presence of divalent metal ions, as monovalent ions (NaCl or KCl) alone showed no activity. However, a screen of Irving–Williams divalent metals demonstrates that NGS12-7 can recruit Ca^2+^, Mn^2+^, or Mg^2+^ as divalent metal ion cofactors. For Ca^2+^- and Mn^2+^-dependent cleavage, saturation occurred at concentrations of <10 mM divalent metal ion, indicating that these ions bind more tightly to the catalytic motif than magnesium. The *k*_obs_ was 0.021 ± 0.0005 min^−1^ in the presence of 10 mM CaCl_2_ and 0.055 ± 0.0008 min^−1^ in the presence of 2 mM MnCl_2_ (Fig. 4c, d).

Next, we examined the potential for NGS12-7 to function as a general RNA-cleaving FANA enzyme. Substitution of the ribo-G residue at the G-U junction with A, U, or C led to a complete loss of activity (Supplementary Fig. 8), indicating that NGS12-7 is specific for RNA substrates with a G-U cleavage site. We then constructed engineered versions of NGS12-7 in which the substrate-binding arms were modified to recognize different RNA substrates (Fig. 4e). Activity was undiminished when substitutions were made to the 5’ and 3’ ends of the original substrate (S1 versus S2 and S3). However, reduced activity was observed for substrates S4 and S5 in which the binding arms flanking the cleavage site were changed to unrelated sequences (Fig. 4f, Supplementary Fig. 9). Changing the divalent metal ion in the cleavage buffer from 25 mM MgCl_2_ to 10 mM CaCl_2_ or 2 mM MnCl_2_ produced a similar trend, indicating that differences in the thermodynamic stability of the enzyme–substrate complex produced by different Watson–Crick base pairs can affect the rates of catalysis. Similar results have been observed for other RNA-cleaving nucleic acid enzymes and can be overcome by varying the length of the binding arms in the substrate recognition domain^5,44^.

**Fig. 5:**
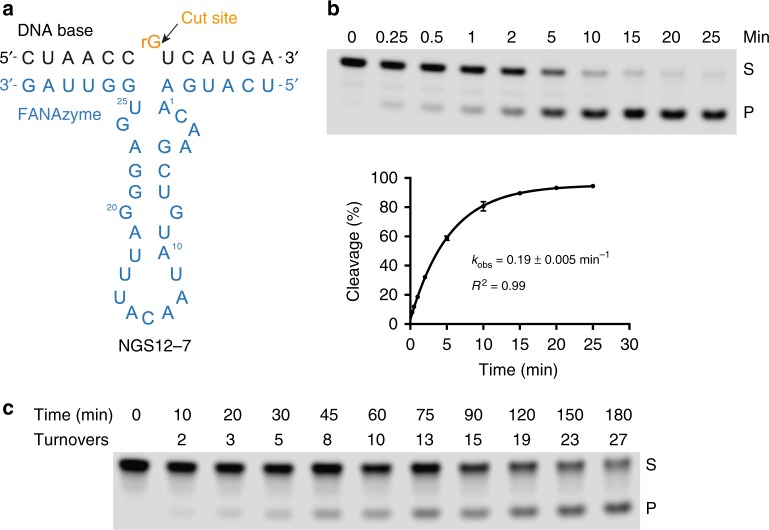
Cleavage of a chimeric DNA/RNA substrate contain a single unpaired ribo-G residue. **a** Predicted secondary structure showing FANAzyme NGS12-7 in complex with the substrate. **b** Substrate cleavage profile under single-turnover conditions. Representative gel image (top) and kinetic profile (bottom). ([Fz] = 2.5 µM, [*S*] = 0.5 µM) *n* = 3, error bars represent s.d. **c** Substrate cleavage under multiple-turnover conditions. ([Fz] = 10 nM, [*S*] = 0.5 µM). All reactions were carried out in trans in 50 mM CHES (pH 8.5) containing 200 mM NaCl and 25 mM MgCl_2_ at 24 °C. S substrate, P cleavage product

Finally, we investigated the ability of NGS12-7 to recognize a DNA substrate containing a single ribo-G residue at the cleavage site (Fig. 5a). All of the experiments performed thus far involved the use of RNA substrates that adopt an A-form helical geometry in the substrate-binding domain; however, a DNA substrate would presumably change this geometry to a B-form helical structure. We therefore tested this possibility by replacing the normal RNA substrate with an DNA substrate that contains a ribo-G nucleotide at the cleavage site. Surprisingly, the pseudo first-order rate constant for chimeric DNA substrate was 7-fold faster (*k*_obs_ ~ 0.2 min^−1^) than the all-RNA substrate (Fig. 5b, c), even though the enzyme was evolved for RNA-cleavage activity. This result suggests that the DNA substrate provides a more favorable geometry for in-line attack on the phosphodiester linkage than the RNA substrate, which could be useful for certain biotechnology applications^45^.

## Discussion

Following the discovery of XNA aptamers by in vitro selection^11,12^, researchers sought to develop the first examples of XNA enzymes that could fold themselves into shapes with catalytic activity. This work was motivated by a longstanding desire to understand the question of what was the first genetic polymer of life on Earth as well as the aspiration to produce catalytic XNAs that could modulate the functional properties of biological RNAs or perform chemical reactions that are not accessible to natural genetic polymers^46,47^. Historically, these questions have been difficult to answer due to the absence of polymerases that are needed to evolve functional XNAs in a test tube. However, with recent advances in polymerase engineering, it is becoming increasingly possible to explore the functional properties of different XNA systems by in vitro selection^48–50^.

In 2015, Holliger and coworkers made an important advance in this area by evolving four different types of artificial genetic polymers (ANA, FANA, HNA, and CeNA) that functioned as catalysts^17^. However, despite the use of elevated reaction conditions (50 mM MgCl_2_ at pH 8.5), the enzymes produced from these selections functioned with only weak catalytic activity. The most proficient catalyst, a FANAzyme with ribonuclease activity, was isolated after 17 rounds of selection from libraries that derived from the high activity 10–23 and 8–17 RNA-cleaving DNA enzyme motifs^4^. This particular enzyme exhibited a catalytic rate (*k*_obs_) of 0.02 min^−1^, which is slower than comparable DNA and RNA enzymes^38–40^. Other XNAzymes discovered in their selections functioned with much lower rates, including some as slow as 0.0001 min^−1^. Moreover, none of the enzymes were shown to function with Michaelis–Menten kinetics, suggesting that these enzymes may not exhibit standard substrate saturation profiles typical of most natural enzymes.

Holliger’s observations, though insightful, raised an important question about the ability of XNAs to adopt well-folded catalytic motifs. Indeed, one could imagine that purely chemical constraints, such as the presence of an unnatural sugar-phosphate backbone, might preclude the ability for certain XNAs to fold into shapes that can properly stabilize the transition state of a given chemical reaction. Such physical constraints could, for example, explain the dominance of ribofuranosyl nucleic acid polymers in biology or the emergence of the RNA world^51^. However, it is also possible that these XNAzymes were constrained by their starting library or the polymerases used to generate them. Indeed, it is generally well understood that most XNA polymerases function with reduced fidelity relative to their natural counterparts^11^.

The current study aimed to advance these earlier findings by determining whether XNAs were capable of folding into well-structured catalytic motifs that functioned with Michaelis–Menten kinetics. The selection and resulting catalysts distinguished themselves in several ways from the previous study^17^. First, a new replication system was developed that relies on natural polymerases and commercial reagents to transcribe and reverse transcribe FANA molecules with high fidelity. This invention opens the world of XNA evolution to researchers who did not previously have access to XNA substrates or engineered polymerases. Second, the selection was initiated from an unbiased random sequence library that was not constrained by a previously discovered catalytic motif. Third, the best FANAzyme was shown to function with Michaelis–Menten kinetics, which produced a maximum rate constant (*k*_cat_) of ~0.2 ± 0.01 min^−1^ and a *K*_M_ of ~600 ± 77 nM in reaction buffer containing 25 mM MgCl_2_ (pH 8.5). These values are similar to many natural and in vitro selected RNA-cleaving ribozymes and deoxyribozymes^38–40^. Fourth, the resulting FANAzyme adopts a well-evolved active fold that is tunable by changing the sequence and length of the substrate-binding domain. This result reflects a strong *K*_M_ value, which is the intrinsic substrate-binding affinity of the enzyme for a target RNA sequence. Last, the catalytic activity itself can be further increased by adjusting certain reaction parameters, including divalent ion concentrations and substrate targets.

Moving forward, it will be interesting to see how far XNA enzymes can be evolved, both in terms of not only catalytic activity but also reaction repertoire. Establishing new examples of XNA enzymes will not only help us to understand the uniqueness of RNA as a prebiotic molecule in the evolution of life^52^ but could also provide a rich new source of biologically stable catalysts that can be used to support diagnostic and therapeutic applications in emerging areas of biomedical research^53^. For this vision to be realized, new XNA polymerases are needed that can faithfully replicate XNA polymers with diverse backbone structures^54^. Even more challenging would be the development of engineered polymerases that can be evolved to recognize new types of XNA polymers that have not yet been the study of chemical synthesis or directed evolution. Such efforts would greatly expand the chemical space of evolvable non-natural genetic polymers for synthetic genetics^55^.

In summary, our work establishes a strategy for the replication and evolution of FANA using natural polymerases and commercially available reagents. The capacity for FANA to fold into shapes with strong catalytic activity shows that natural genetic polymers are not unique in their ability to function as robust nucleic acid enzymes. The methodology developed here, coupled with the chemical and biological stability of FANA polymers, provides access to nuclease-resistant aptamers and catalysts for a broad range of problems in molecular medicine, biotechnology, and material science.

## Methods

### General information

2’F-araNTPs (faATP, faCTP, faGTP, faUTP) were obtained from Metkinen Chemistry (Kuusisto, Finland). FANA phosphoramidites were purchased from Glen Research (Sterling, Virginia). FANA oligonucleotides were synthesized on an ABI3400 DNA synthesizer using chemical synthesis reagents purchased from Glen Research (Sterling, Virginia). Powdered dNTPs were purchased from Sigma-Aldrich (St. Louis, MO). DNA oligonucleotides were purchased from Integrated DNA Technologies (Coralville, IA), purified by denaturing polyacrylamide gel electrophoresis (PAGE), and quantified by ultraviolet (UV) absorbance. All oligonucleotides used in this study are provided in Supplementary Table 1. YM-10 microcentrifugal concentrators were purchased from EMD Millipore (Billerica, MA). LC Green Plus fluorescent dye was obtained from BioFire Defense (Salt Lake City, Utah). ThermoPol buffer, Taq DNA polymerase, *G. stearothermophilus* Bst DNA polymerase, LF, and its variants Bst 2.0 DNA polymerase (2.0), and Bst 3.0 DNA polymerase (3.0), DH5α competent cells, and Monarch DNA gel extraction kits were purchased from New England Biolabs (Ipswich, MA). 3′–5′ exonuclease-deficient (exo−) archaeal polymerases isolated from *Thermococcus* sp. 9°N (9°N), *Pyrococcus* sp. deep vent (DV), *T. gorgonarius* (Tgo), *T. kodakarensis* (Kod), and *G. stearothermophilus* Bst DNA polymerase, LF* were expressed and purified from *E. coli* of the XL1-Blue strain from Agilent Technologies (Santa Clara, CA) as previously described^1^. TOPO Cloning Kit was purchased from ThermoFisher Scientific (Waltham, MA).

### Polymerase purification

Polymerases were expressed and purified as reported previously^1^. Briefly, XL1-blue *E. coli* cells carrying custom pGDR11 polymerase expression plasmids were inoculated in 1 L of LB-ampicillin (100 μg mL^−1^) liquid medium and grown at 37 °C with shaking at 225 rpm. At OD600 = 0.6, the expression culture was cooled to 15 °C and induced with IPTG at a final concentration of 0.5 mM and incubated overnight at 15 °C with shaking at 225 rpm. Cells were harvested by centrifugation for 20 min at 3315 × *g* at 4 °C and lysed in 40 mL buffer (10 mM Tris pH 8.0, 500 mM KCl, 10% glycerol) by sonication on ice. The cell lysate was centrifuged for 30 min at 23,708 × *g* at 4 °C, and the clarified supernatant was heat for 30 min at 80 °C, then immediately cooling for 30 min on ice. The lysate was clarified again by centrifugation for 20 min at 23,708 × *g* at 4 °C. Nucleic acids were precipitated by adding 10% (v/v) polyethyleneimine to a final concentration of 0.5% and incubating for 30 min on ice, then centrifuging for 30 min at 23,708 × *g* at 4 °C. The supernatant was recovered, and the polymerase was precipitated by adding 60% (w/v) ammonium sulfate, incubating for 30 min on ice, and then centrifuging for 30 min at 23,708 × *g* at 4 °C. Protein pellets were suspended in 4 °C buffer (10 mM Tris pH 8.0, 50 mM KCl, 10% glycerol). Particulates were removed by centrifuging for 10 min at 23,708 × *g* at 4 °C. Polymerases were then purified by 5 mL heparin high-performance (HP) affinity chromatography with step elutions of 100, 250, 500, and 1000 mM KCl. Fractions corresponding to protein of the correct size were verified by sodium dodecyl sulfate–PAGE, combined, quantified by UV absorbance at 280 nm, and stored at 4 °C.

### Polymerase screen for FANA synthesis activity

Polymerase activity assays were performed in 10 µL reaction volumes containing 1 µM of primer-template complex, 1 µM of polymerase (9°N, DV, Kod, or Tgo), and 100 µM of each 2’F-araNTP in 1× ThermoPol buffer [20 mM Tris-HCl, 10 mM (NH_4_)_2_SO_4_, 10 mM KCl, 2 mM MgSO_4_, 0.1% Triton X-100, pH 8.8]. DNA primer IR800-PBS8-short was used for reactions performed on the 30-mer DNA template and the IR800-PBS9 primer was used for reactions performed with the L16 DNA library. The primer-template complex was annealed in 1× ThermoPol buffer by heating for 5 min at 90 °C and cooling for 10 min at 4 °C. Primer-extension reactions were performed for 1 h at 55 °C with the L16 library, and two time points (15 and 30 min) were obtained by quenching 3 µL of the primer extension reaction with 30 µL (10 equivalents, v/v) of formamide stop buffer (99% deionized formamide, 25 mM EDTA) for reactions with the 30-mer DNA template. Owing to the elevated stability of the chimeric DNA/FANA heteroduplex, samples were denatured for 15 min at 95 °C before analyzing by denaturing PAGE. Gels were visualized using a LI-COR Odyssey CLx imager.

### Reverse transcription of FANA to DNA

Reverse transcription reactions were performed using either a chemically synthesized 30mer FANA oligonucleotide template or a FANA version of the L16 library obtained by transcribing the L16 DNA library containing N40 random region (N = A:T:C:G = 35:35:15:15) into FANA. The FANA library was transcribed with Tgo DNA polymerase for 3 h at 55 °C by extending 1 µM of the PEGylated PBS9 DNA primer (IR800-PEG-PBS9) annealed to 1 µM of the L16 DNA library in a 1 mL reaction (see FANA transcription reaction for details). The transcribed FANA library was PAGE purified, electroeluted, exchanged into H_2_O using EMD Millipore YM-10 microcentrifugal device, and UV quantified. FANA reverse transcriptions were performed in 10 µL reaction volumes containing 1 µM of primer-template complex, 1x ThermoPol buffer supplemented with 3 mM MgCl_2_, 500 µM of each dNTP, and either 0.8 U/µL of commercial Bst DNA polymerase or 1 µM of *E. coli* expressed Bst DNA polymerase (LF*). DNA primer IR800-PBS8-short was used for reactions performed on the 30mer FANA template and the IR800-PBS7 primer was used for reactions performed with the L16 FANA library. The reactions were incubated for 30 min with the 30mer template and 3 h with the L16 random library at 50 °C. Following incubation, reactions were quenched using 100 µL (10 equivalents, v/v) of formamide stop buffer (99% deionized formamide, 25 mM EDTA). Reaction products were denatured for 15 min at 95 °C and analyzed by 10–20% denaturing PAGE. Gels were visualized using a LI-COR Odyssey CLx.

### Measurement of catalytic rates of Tgo and Bst LF*

Kinetic measurements were performed in 96-well format as previously described.^2^ Each measurement (10 μL) contained 1 μM of the self-priming 30-mer hairpin template (30-mer HP), 1× ThermoPol buffer, 100 μM of each nucleotide triphosphate, 2× LC Green Plus fluorescent dye, and DNA polymerase (10 nM Tgo or 1 μM Bst LF*). Bst LF* reverse transcription reactions were supplemented with 3 mM MgCl_2_. Reactions were denatured for 2 min at 95 °C and extended for 1 h at 55 °C (for Tgo) or 50 °C (for Bst LF*) with fluorescence intensity recorded at 6-s intervals. Fluorescence data collected from the first 18 s were excluded to eliminate possible artifacts caused by temperature equilibration. Fluorescence data for each reaction were normalized by subtracting baseline fluorescence and dividing by the difference between minimum and maximum fluorescence values taken from the min. and max. standards. Fluorescence was converted to nucleotides per polymerase before using the conversion factor *F*_max_ (*F*_max_ = *C*_n_ × *n* × *C*_p_^−1^, where *F*_max_ is the maximum fluorescence for the max. standard, *C*_n_ is the concentration of the hairpin template, *n* is the number of incorporated nucleotides, and *C*_p_ is the concentration of polymerase). Kinetic values were determined by performing a linear regression of nucleotides per polymerase over reaction time. The reported values were derived from a representative plot taken from at least three independent replicates.

### Measurement of FANA replication fidelity

FANA replication fidelity was measured by sequencing a DNA template that was transcribed into FANA and reverse transcribed back into cDNA. For FANA transcription (500 pmol), 1 µM of PBS8_extra primer containing a single nucleotide mismatch in the base pairing region was annealed with 1 µM of the 4NT9G template in 1× ThermoPol buffer by heating for 5 min at 90 °C and cooling for 10 min at 4 °C. The primer-template complex was then combined with 100 µM of each 2’F-araNTP, 1 µM Tgo DNA polymerase, and incubated for 3 h at 55 °C in a final volume of 500 µL. The fully extended product was purified by denaturing PAGE (8 M urea), electroeluted, and desalted using EMD Millipore YM-10 microcentrifugal concentrators. Next, the FANA product was reverse transcribed back into DNA using 1 µM Bst LF* DNA polymerase (expressed from *E. coli*). The reaction (20 µL volume) contained 1× ThermoPol buffer supplemented with 3 mM MgCl_2_, 1 µM of PBS7 primer, and 500 µM of each dNTP. After heating for 3 h at 50 °C, the reaction was treated with 0.8 U of proteinase K for 20 min at 50 °C, then held for 10 min at 95 °C. The cDNA was PCR amplified using the extra/PBS7 primer pair, agarose purified, ligated into a TOPO vector, and cloned into *E. coli* DH5α cells. Individual colonies were grown in liquid media and sequenced using the M13R primer by Retrogen (San Diego, CA). DNA sequences were aligned with the starting 4NT9G template and analyzed for point mutations using MEGA7.

### In vitro selection of RNA-cleaving FANA catalysts

The 5’-biotinylated selection primer containing the all-RNA substrate was generated by ligating the 5’-biotinylated fragment 1 (Biotin-fragment I) and the 5’-phosphorylated fragment 2 (AllRNA-fragment II) containing the all-RNA substrate and an internal fluorescein label using T4 DNA ligase in the presence of 2 equivalents of a DNA splint (Splint) for 2 h at room temperature following a standard ligation protocol. The ligation product was purified by denaturing PAGE, electroeluted, desalted using EMD Millipore YM-10 microcentrifugal concentrators and UV quantified. For each round of selection, 1 nmol of DNA library was transcribed into FANA by incubating for 2 h at 55 °C in a 1 mL reaction volume. The reaction contained 1 µM selection primer-DNA library duplex, 1× ThermoPol buffer, 1 µM RNA blocker oligonucleotide (RNA blocker), and 100 µM of each 2’F-araNTP and 1 µM Tgo DNA polymerase. Following incubation, the reaction was quenched with enough 0.5 M EDTA (pH 8.0) to give a final concentration of 25 mM.

Five hundred μL of streptavidin-coated magnetic beads were washed 3× with 500 μL of wash buffer (50 mM Tris-HCl, pH 7.5, 200 mM NaCl, and 1 mM EDTA). The EDTA quenched primer extension product was incubated with the streptavidin beads for 20 min at room temperature to immobilize the FANA–DNA duplex on the beads. Following two more washes with 500 μL of wash buffer, the template strand was removed by five quick wash steps (<30 s per wash) with 500 μL of cold NaOH (0.1 M) containing 1 mM EDTA. The bound FANA strand was immediately neutralized using 1 mL of neutralization buffer (50 mM Tris-HCl, pH 6.0, 1 mM EDTA), followed by a final 1 mL wash with nuclease-free H_2_O. The FANA library was then incubated in 100 μL of cleavage buffer (50 mM Tris-HCl, pH 7.5, 200 mM NaCl, 20 mM MgCl_2_). For the initial round of selection, the library was incubated in cleavage buffer containing 20 mM MgCl_2_ for 20 h at room temperature (23 °C). For the subsequent rounds, the MgCl_2_ concentration in cleavage buffer was gradually reduced to 2 mM and the incubation time was shortened to 0.5 h.

Following incubation with MgCl_2_, a magnetic field was applied to the beads and the supernatant (90 μL) was recovered, desalted by exchanging into 500 μL of H_2_O, and concentrated to the final volume of 11.5 μL using EMD Millipore YM-10 microcentrifuge concentrators. The population of FANAzymes was reverse transcribed into cDNA by incubating for 3 h at 50 °C with Bst LF*. The reaction (20 µL) contained 1× ThermoPol buffer supplemented with 3 mM of MgCl_2_, 1 µM of reverse transcription primer PBS7, and 500 µM of each dNTP. The cDNA was amplified by PCR using Taq DNA polymerase with the PBS11/PBS7 primer pair: 95 °C for 8 min, *N* cycles of (95 °C for 25 s; 58 °C for 15 s; 72 °C for 30 s). The number of cycles (*N*) was optimized for each round of selection by sampling PCR reactions every other cycle up to 20 cycles. The amplified DNA was used as template for a second PCR reaction in which PBS11 was replaced by PEGylated PBS11 (PEG-PBS11) following PCR procedures described above. The second PCR product was purified by denaturing PAGE, and the non-PEGylated strand was used as template for the next round of selection. After 12 rounds of selective amplification, cDNA amplicons were cloned into *E. coli* DH5α cells for Sanger sequencing by Retrogen (San Diego, CA).

### Illumina NGS

Round 12 cDNA amplicons were generated by PCR using Taq DNA polymerase. Polyclonal cDNA amplicons were purified by 2% agarose using the Monarch DNA Gel Extraction Kit. The cDNA amplicons were then made into barcoded Illumina libraries using the Apollo 324 platform and PrepX ILM DNA Kit and protocol (Wafergen Bio-Systems, Fremont, CA). The barcoded libraries were spiked into a multiplex of Illumina libraries. The multiplex was denatured and clustered at 12 pM for sequencing on a HiSeq 2500 (Illumina, San Diego, CA) in rapid run mode (8 million reads per amplicon library, single-end 100 cycles) by UCI Genomics High Throughput Facility. Data were analyzed on UCI HPC (https://hpc.oit.uci.edu) using in-house scripts, and sequences were ordered by abundance.

### FANAzyme preparation

FANAzymes were enzymatically transcribed in 1× ThermoPol buffer containing 1 µM of primer-template complex, 1 µM of Tgo, and 100 µM of each 2’F-araNTP. A synthetic DNA template containing (AAC)_7_ repeats at the 3’-end designed for strand separation and a 16-mer PBS11 primer were annealed in ThermoPol buffer by heating for 5 min at 90 °C and cooling for 10 min at 4 °C. FANA transcription was initiated by the addition of 2’F-araNTPs and Tgo. Reactions were incubated for 1 h at 55 °C and quenched with 0.5 M EDTA (pH 8.0) to a final concentration of 25 mM and lyophilized dry. Equal volume of formamide stop buffer (99% deionized formamide, 25 mM EDTA) was used to resuspend the dry pellet followed by heat denaturation for 15 min at 95 °C. After cooling for 5 min on ice, the sample was resolved by 10% denaturing purification PAGE (8 M urea), and the gel was visualized by UV-shadowing. Full-length FANA transcript was excised, electroeluted, exchanged into H_2_O using EMD Millipore YM-3 microcentrifugal device, and UV quantified by Nanodrop.

### FANAzyme reactions under single-turnover conditions

Single-turnover reactions were conducted in 50 mM CHES buffer (pH 8.5) containing 200 mM NaCl, 25 mM MgCl_2_, 0.5 μM of substrate, and 2.5 μM of FANAzyme at 23 °C. Purified FANAzymes and RNA substrates were annealed in 50 mM CHES buffer by heating for 5 min at 90 °C and cooling for 5 min on ice. Reactions were initiated by the addition of NaCl and MgCl_2_ to the reaction. For determination of pseudo first-order rate constant, multiple time points were collected by quenching 1.5 μL of reaction using 15 μL (10 equivalents, v/v) of formamide stop buffer (99% deionized formamide, 25 mM EDTA) and cooling on ice. Samples were denatured for 15 min at 95 °C and analyzed by 15% denaturing PAGE. Gels were visualized and quantified using a LI-COR Odyssey CLx. Values of *k*_obs_ were calculated by fitting the percentage of substrate cleaved and reaction time (min) to the first-order decay Eq. (1) using Prism 6 (GraphPad, USA):

1 $$P_t = P_\infty \left( {1 - e^{ - k_{{\mathrm{obs}}}t}} \right)$$

where *P*_*t*_ is the percentage of cleaved substrate at time *t*, *P*_*∞*_ is the apparent reaction plateau, and *k*_obs_ is the observed first-order rate constant.

The pH titration experiments used the following buffers: 50 mM HEPES (pH 7.0), Tris-HCl (pH 7.5 and 8.0), CHES (pH 8.5–10.0), or CAPS (pH 10.25 and 10.5). In Mg^2+^ concentration titration experiments, MgCl_2_ was added to final concentrations of 0, 5, 10, 25, 50, 100, 200, 400, 600, 800, and 1000 mM. In both cases, time points were obtained at 0 and 15 min by quenching 1.5 μL of reactions using 15 μL (10 equivalents, v/v) of formamide stop buffer (99% deionized formamide, 25 mM EDTA) and cooling on ice. In the screen for metal ion requirement experiments, MgCl_2_ was substituted by 10 mM of CaCl_2_, or ZnCl_2_, or CuCl_2_, or CoCl_2_, or NiCl_2_, or divalent metal ion was totally depleted but with only 1 M NaCl or KCl presenting in the 50 mM CHES buffer (pH 8.5). For each metal ion screen reaction, two time points were obtained, at 0 and 60 min, by quenching 1.5 μL of reactions using 15 μL (10 equivalents, v/v) of formamide stop buffer (99% deionized formamide, 25 mM EDTA) and cooling on ice.

Similar to the Mg^2+^ concentration titration experiments, MnCl_2_ or CaCl_2_ was titrated at concentrations of 50 μM, 100 μM, 200 μM, 500 μM, 1 mM, 2 mM, 5 mM, and 10 mM in 50 mM CHES (pH 8.5) supplemented with 200 mM NaCl. Two time points were obtained at 0 and 60 min for each concentration as described above. Values of *k*_obs_ were only measured when Mg^2+^ was substituted by 2 mM MnCl_2_ or 10 mM CaCl_2_ as described above.

### FANAzyme reactions under multiple-turnover conditions

Multiple-turnover cleavage assays were performed in 50 mM CHES buffer (pH 8.5) containing 200 mM NaCl, 25 mM MgCl_2_, 10 nM of FANAzyme (NGS12-7), and 8 substrate concentrations (100, 200, 300, 400, 500, 800, 1000, and 2000 nM) that were in at least 10-fold excess of NGS12-7 and exceeding *K*_M_ at 23 °C. The reaction rate (*v*_obs_) for each substrate concentration ([*S*]) was calculated by a linear fit of at least five data points obtained over the first 10–15% of cleavage reaction. *k*_cat_ and *K*_M_ values were determined by plotting the *v*_obs_ values versus [*S*] to the Michaelis–Menten Eq. (2):

2 $$\frac{{v_{obs}}}{{[E]}} = k_{{\mathrm{cat}}}\frac{{\left[ S \right]}}{{K_{\mathrm{M}} + \left[ S \right]}}$$

where [*E*] represents the FANAzyme concentration, which was 10 nM in the assays here.

### Analysis of 5’-RNA cleavage product by high-resolution MS

A version of 5’-FAM-labeled RNA substrate containing T_24_ DNA tail at the 3’-end that was designed to ensure sufficient separation of the desired 5’-RNA cleavage product (7 mer) from 3’-cleavage product (30 mer) or the unreacted intact RNA substrate (37 mer) on purification PAGE was used in the preparative scale (500 μL) single-turnover cleavage reaction. As described above, 500 μL of cleavage reaction in 50 mM CHES buffer (pH 8.5) containing 200 mM NaCl, 25 mM MgCl_2_, 0.5 μM of substrate, and 2.5 μM of FANAzyme was incubated for 60 min at 23 °C before quenching using 0.5 M EDTA (pH 8.0) to final concentration of 25 mM. The quenched reaction was lyophilized dry, resuspended in 500 μL of formamide stop buffer (99% deionized formamide, 25 mM EDTA), denatured for 15 min at 95 °C, and resolved by 15% denaturing PAGE. Gel samples were visualized by UV-shadowing, and only the 5’-RNA cleavage product was excised, electroeluted, exchanged into H_2_O using EMD Millipore YM-3 microcentrifugal device, and UV quantified by Nanodrop. The sample was then submitted to Novatia (Newtown, PA) for high-resolution exact mass determination using ESI.

## Electronic supplementary material

Supplementary Information

## Data Availability

All relevant data are contained within the manuscripts or are available from the authors.
