# Supplementary material for: Evolution of a General RNA-Cleaving FANA Enzyme
Source: Nat Commun. 2018 Nov 29;9:5067. doi: 10.1038/s41467-018-07611-1 (PMC6265334; doi:10.1038/s41467-018-07611-1)
Supplement: Supplementary file 1 — Supplementary Information [file 41467_2018_7611_MOESM1_ESM.pdf]

SUPPLEMENTAL INFORMATION

Evolution of a General RNA-Cleaving FANA Enzyme

Yajun Wang, Arlene K. Ngor, Ali Nikoomanzar, and John C. Chaput*

Departments of Pharmaceutical Sciences, Chemistry, and Molecular Biology and Biochemistry. University of California, Irvine, CA 92697-3958.

Table of contents

List of Supplementary Figures

Figure S1. FANA synthesis and reverse transcription of random-sequence libraries
Figure S2. FANA replication fidelity mediated by Tgo and Bst LF*
Figure S3. FANA library assembly strategy
Figure S4. Selection parameters
Figure S5. Representative PAGE showing the single-turnover cleavage activity of NGS12-1 and NGS12-7
Figure S6. PAGE gel image showing the cleavage activity of NGS12-7 DNA version.
Figure S7. High-resolution electrospray ionization (ESI) mass spectrum of the upstream fragment of the RNA cleavage product
Figure S8. Nucleoside preference at the unpaired residue in the RNA substrate
Figure S9. Cleavage of substrate S-4 and S-5 in the absence of NGS12-7
Figures S10-S15. Uncropped images of gels in main text figures

List of Supplementary Tables

Table S1. Table of oligonucleotides
Table S2. Functional analysis of FANA sequences identified by Sanger sequencing
Table S3. Functional analysis of the most abundant NGS sequences
Table S4. Highly abundant NGS sequences with homology to NGS12-1

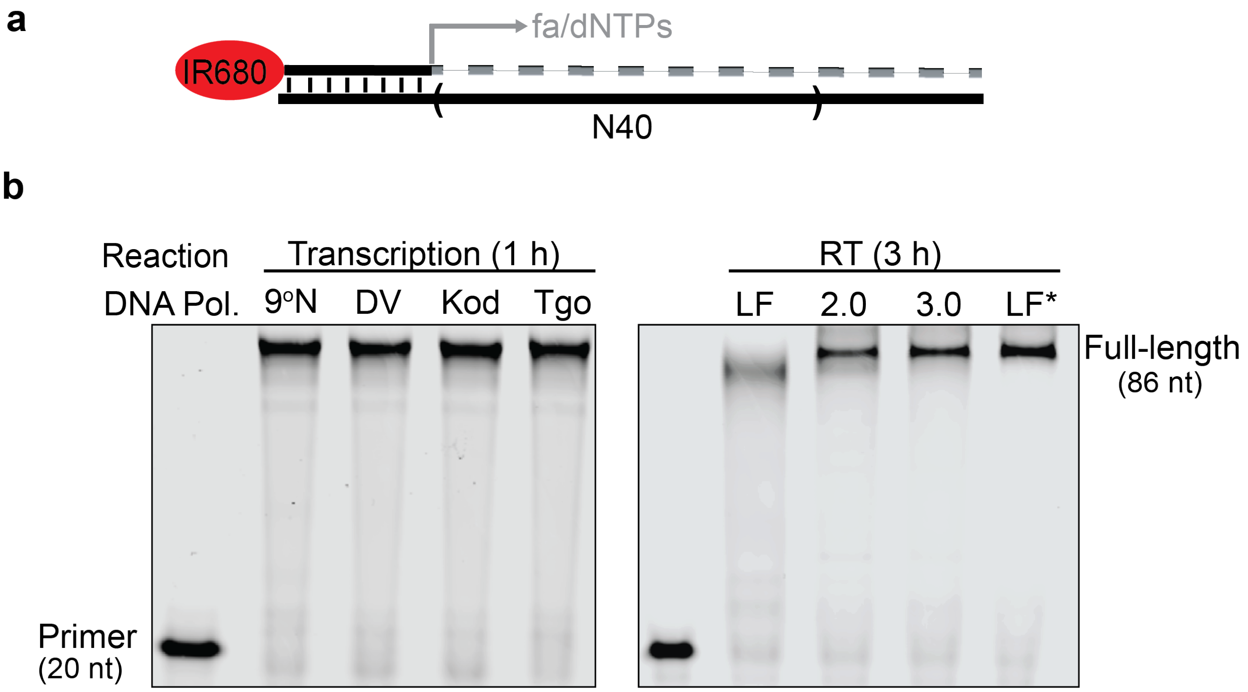

Supplementary Figure 1. FANA synthesis and reverse transcription of random-sequence libraries. (**a**) Schematic representation of each primer extension assay. (**b**) FANA transcription activity of wild-type (exo-) 9°N, DV, Kod, and Tgo (left panel) and FANA reverse transcription activity of Bst polymerase LF, 2.0, 3.0, and LF* (RT, right panel).

a

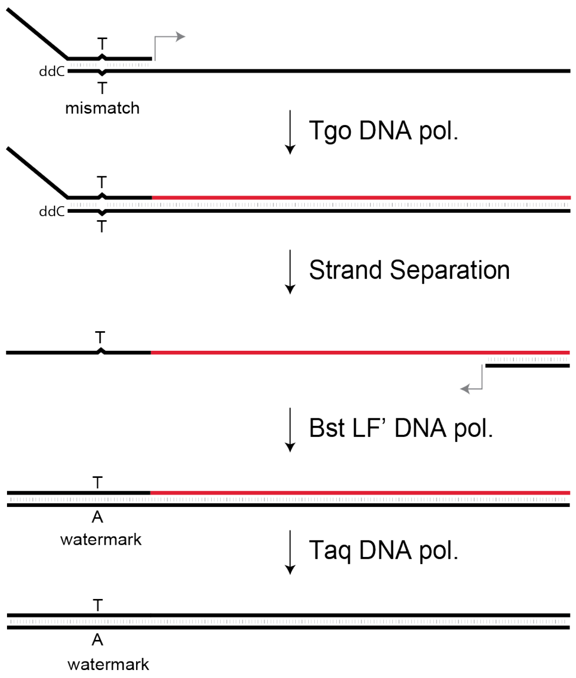

b

4NT9G	T	T	A	A	G	A	C	T	C	G	C	C	A	T	G	T	T	A	C	G	A	T	C	T	G	C	C	A	A	G	T	A	C	A	G	C	C	T	T	G	A	A	T	C	G	T	C	A	C	T
FANA-Tgo wt-1	.	.	.	.	.	.	.	.	.	.	.	.	.	.	.	.	.	.	.	.	.	.	.	.	.	.	.	.	.	.	.	.	.	.	.	.	.	.	.	.	.	.	.	.	.	.	.	.	.	.
FANA-Tgo wt-2	.	.	.	.	.	.	.	.	.	.	.	.	.	.	.	.	.	.	.	.	.	.	.	.	.	.	.	.	.	.	.	.	.	.	.	.	.	.	.	.	.	.	.	.	.	.	.	.	.	.
FANA-Tgo wt-3	.	.	.	.	.	.	.	.	.	.	.	.	.	.	.	.	.	.	.	.	.	.	.	.	.	.	.	.	.	.	.	.	.	.	-	.	.	.	.	.	.	.	.	.	.	.	.	.	.	.
FANA-Tgo wt-4	.	.	.	.	.	.	.	.	.	.	.	.	.	.	.	.	.	.	.	.	.	.	.	.	.	.	.	.	.	.	.	.	.	.	.	.	.	.	.	.	.	.	.	.	.	.	.	.	.	.
FANA-Tgo wt-17	.	.	.	.	.	.	.	.	.	.	.	.	.	.	.	.	.	.	.	.	.	.	.	.	.	.	.	.	.	.	.	.	.	.	.	.	.	.	.	.	.	.	.	.	.	.	.	.	.	.
FANA-Tgo wt-18	.	.	.	.	.	.	.	.	.	.	.	.	.	.	.	.	.	.	.	.	.	.	.	.	.	.	.	.	.	.	.	.	.	.	.	.	.	.	.	.	.	.	.	.	.	.	.	.	.	.
FANA-Tgo wt-24	.	.	.	.	.	.	.	.	.	.	.	.	.	.	.	.	.	.	.	.	.	.	.	.	.	.	.	.	.	.	.	.	.	.	.	.	.	.	.	.	.	.	.	.	.	.	.	.	.	.
FANA-Tgo wt-25	.	.	.	.	.	.	.	.	.	.	.	.	.	.	.	.	.	.	.	.	.	.	.	.	.	.	.	.	.	.	.	.	.	.	.	.	.	.	.	.	.	.	.	.	.	.	.	.	.	.
FANA-Tgo wt-27	.	.	.	.	.	.	.	.	.	.	.	.	.	.	.	.	C	.	.	.	.	.	.	.	.	.	.	.	.	.	.	.	.	.	.	.	.	.	.	.	.	.	.	.	.	.	.	.	.	.
FANA-Tgo wt-29	.	.	.	.	.	.	.	.	.	.	.	.	.	.	.	.	.	.	.	.	.	.	.	.	.	.	.	.	.	.	.	.	.	.	.	.	.	.	.	.	.	.	.	.	.	.	.	.	.	.
FANA-Tgo wt-31	.	.	.	.	.	.	.	.	.	.	.	.	.	.	.	.	.	.	.	.	.	.	.	.	.	.	.	.	.	.	.	.	.	.	.	.	.	.	.	.	.	.	.	.	.	.	.	.	.	.
FANA-Tgo wt-32	.	.	.	.	.	.	.	.	.	.	.	.	.	.	.	.	.	.	.	.	.	.	.	.	.	.	.	.	.	.	.	.	.	.	.	.	.	.	.	.	.	.	.	.	.	.	.	.	.	.
FANA-Tgo wt-33	.	.	.	.	.	.	.	.	.	.	.	.	.	.	.	.	.	.	.	.	.	.	.	.	.	.	.	.	.	.	.	.	.	.	.	.	.	.	.	.	.	.	.	.	.	.	.	.	.	.
FANA-Tgo wt-35	.	.	.	.	.	.	.	.	.	.	.	.	.	.	.	.	.	.	.	.	.	.	.	.	.	.	.	.	.	.	.	.	.	.	.	.	.	.	.	.	.	.	.	.	.	.	.	.	.	.
FANA-Tgo wt-39	.	.	.	.	.	.	.	.	.	.	.	.	.	.	.	.	.	.	.	.	.	.	.	.	.	.	.	.	.	.	.	.	.	.	.	.	.	.	.	.	.	.	.	.	.	.	.	.	.	.
FANA-Tgo wt-42	.	.	.	.	.	.	.	.	.	.	.	.	.	.	.	.	.	.	.	.	.	.	.	.	.	.	.	.	.	.	.	.	.	.	.	.	.	.	.	.	.	.	.	.	.	.	.	.	.	.
FANA-Tgo wt-43	.	.	.	.	.	.	.	.	.	.	.	.	.	.	.	.	.	.	.	.	.	.	.	.	.	.	.	.	.	.	.	.	.	.	.	.	.	.	.	.	.	.	.	.	.	.	.	.	.	.
FANA-Tgo wt-46	.	.	.	.	.	.	.	.	.	.	.	.	.	.	.	.	.	.	.	.	.	.	.	.	.	.	.	.	.	.	.	.	.	.	.	.	.	.	.	.	.	.	.	.	.	.	.	.	.	.
FANA-Tgo wt-48	.	.	.	.	.	.	.	.	.	.	.	.	.	.	.	.	.	.	.	.	.	.	.	.	.	.	.	.	.	.	.	.	.	.	.	.	.	.	.	.	.	.	.	.	.	.	.	.	.	.
FANA-Tgo wt-49	.	.	.	.	.	.	.	.	.	.	.	.	.	.	.	.	.	.	.	.	.	.	.	.	.	.	.	.	.	.	.	.	.	.	.	.	.	.	.	.	.	.	.	.	.	.	.	.	.	.
FANA-Tgo wt-50	.	.	.	.	.	.	.	.	.	.	.	.	.	.	.	.	.	.	.	.	.	.	.	.	.	.	.	.	.	.	.	.	.	.	.	.	.	.	.	.	.	.	.	.	.	.	.	.	.	.
FANA-Tgo wt-52	.	.	.	.	.	.	.	.	.	.	.	.	.	.	.	.	.	.	.	.	.	.	.	.	.	.	.	.	.	.	.	.	.	.	.	.	.	.	.	.	.	.	.	.	.	.	.	.	.	.
FANA-Tgo wt-55	.	.	.	.	.	.	.	.	.	.	.	.	.	.	.	.	.	.	.	.	.	.	.	.	.	.	.	.	.	.	.	.	.	.	.	.	.	.	.	.	.	.	.	.	.	.	.	.	.	.
FANA-Tgo wt-56	.	.	.	.	.	.	.	.	.	.	.	.	.	.	.	.	.	.	.	.	.	.	.	.	.	.	.	.	.	.	.	.	.	.	.	.	.	.	.	.	.	.	.	.	.	.	.	.	.	.

Supplementary Figure 2. **FANA replication fidelity mediated by Tgo and Bst LF***.
(**a**) Schematic representation of the transcription and reverse transcription process used to measure the fidelity of FANA replication. (**b**) Sequence alignment at the DNA level reveals 1 mutation (T-to-C) and 1 deletion (-) from 1200-nt positions. This yields an overall fidelity of ~99.9%.

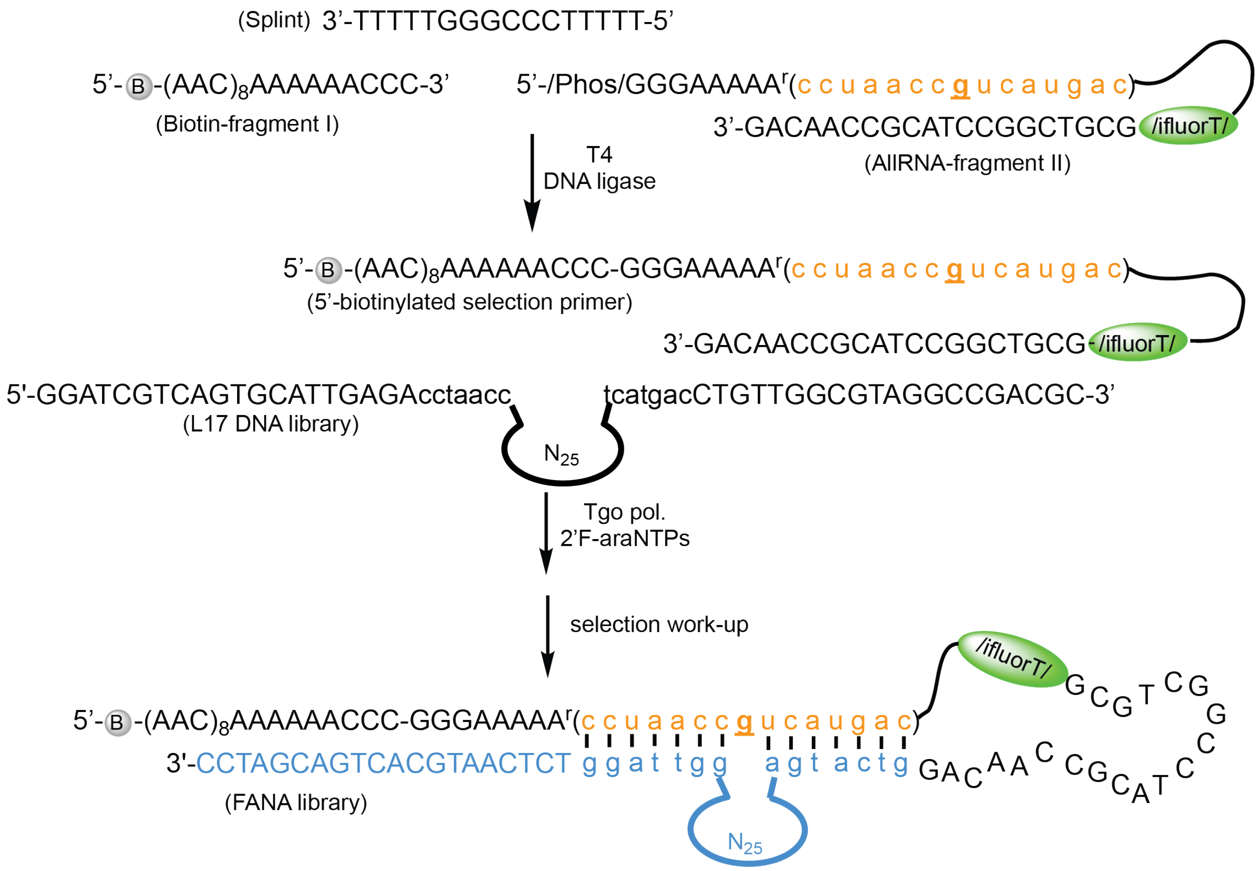

Supplementary Figure 3. FANA library assembly strategy. Starting DNA library assembly and transcription into FANA. Color code: DNA (black), RNA (orange), and FANA (blue). The letters in lower case show the substrate-enzyme binding region. Ribo-G at the cleavage site is underlined and the fluorescent dye is given in green.

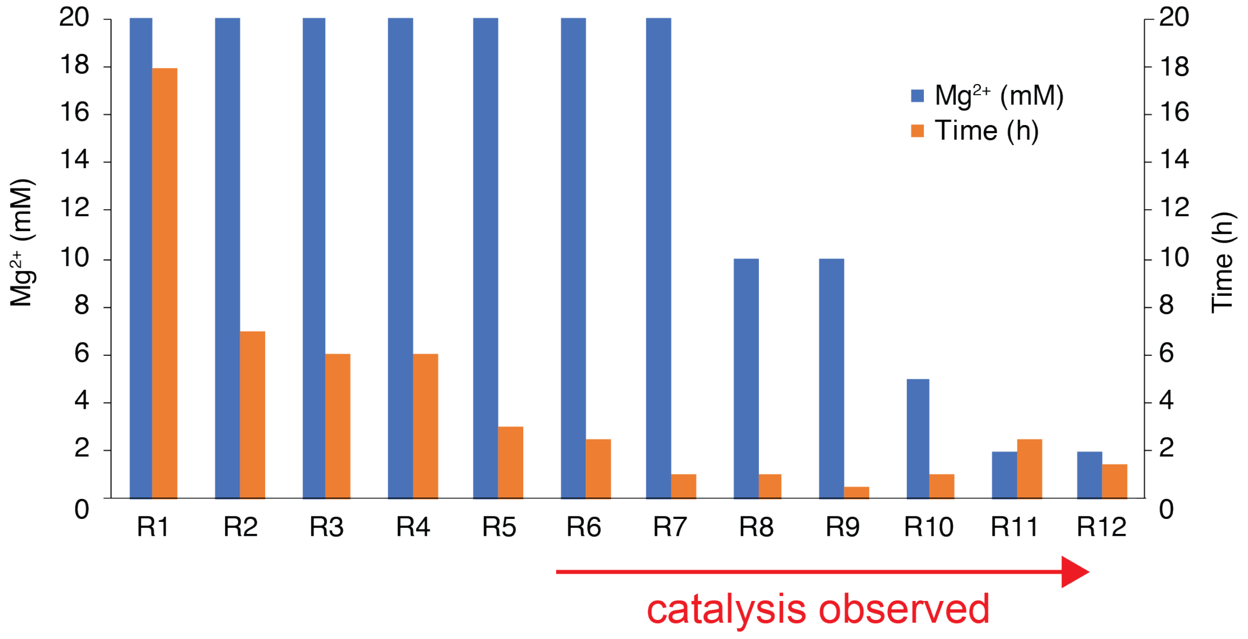

Supplementary Figure 4. **Selection parameters used to isolate functional FANA enzymes**. Over the course of 12 rounds of selection, the Mg^{2+} concentration and reaction time were reduced to favor the enrichment of high activity catalysts. In-gel cleavage activity was observed starting from round 6.

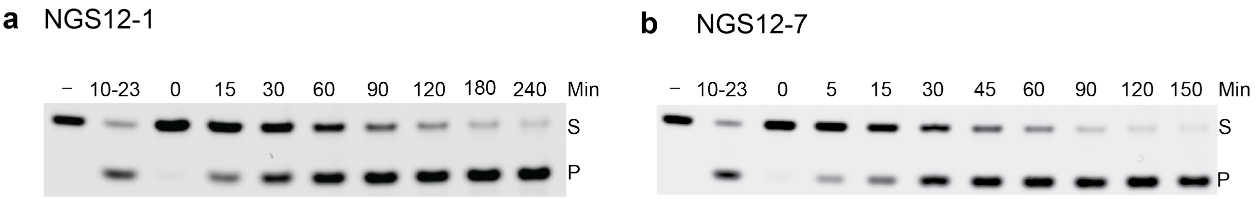

Supplementary Figure 5. **Representative PAGE (8 M urea) images showing the single-turnover cleavage activity of NGS12-1 (a) and NGS12-7 (b)**. Reactions were carried out in trans in 50 mM CHES (pH 8.5) containing 200 mM NaCl and 25 mM $MgCl_2$ at 23 °C under single-turnover conditions ([Fz] = 2.5 μM, [S] = 0.5 μM). S: substrate; P: cleavage product.

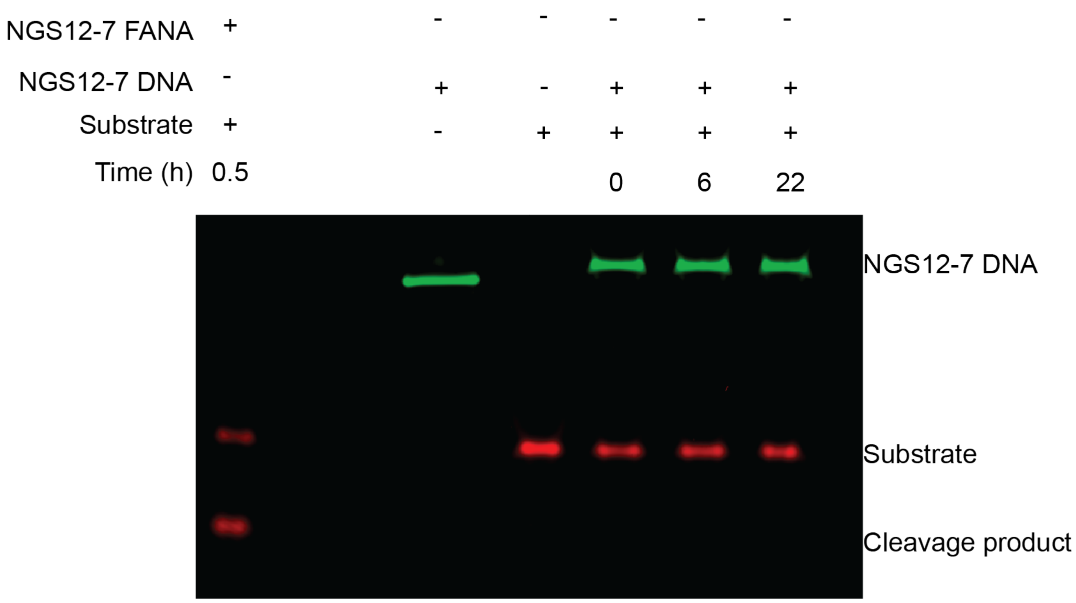

Supplementary Figure 6. **PAGE gel (8 M urea) image showing the cleavage activity of NGS12-7 DNA version.** Reactions were carried out in trans in 50 mM CHES (pH 8.5) containing 200 mM NaCl and 25 mM $MgCl_2$ at 24 °C under single-turnover conditions ([NGS12-7 DNA] = 2.5 uM, [Substrate] = 0.5 uM). No substrate cleavage was observed over 22 h of reaction incubation.

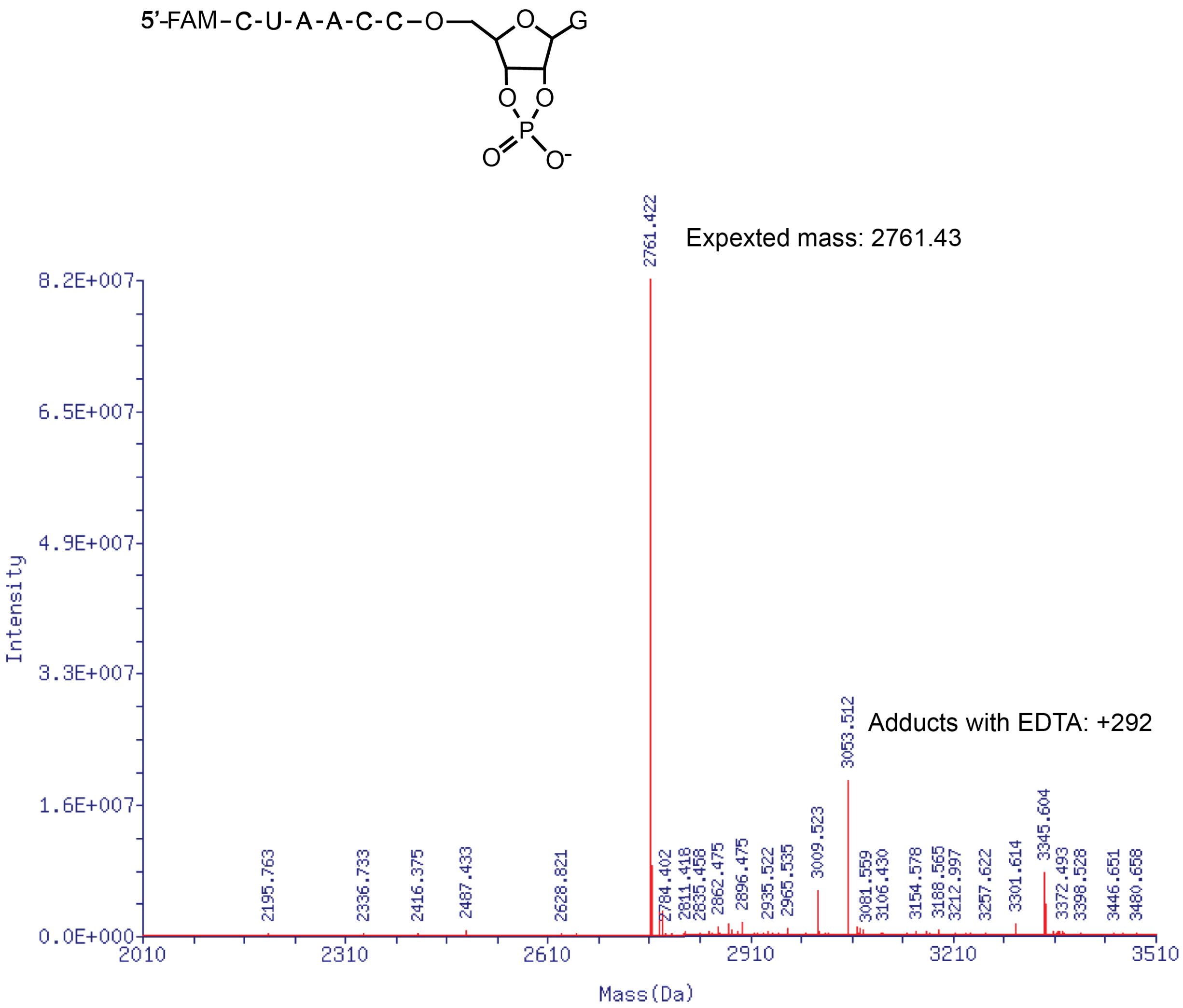

Supplementary Figure 7. **High-resolution electrospray ionization (ESI) mass spectrum of the upstream fragment of the RNA cleavage product**. ESI spectrum of PAGE purified 5'-cleavage product shows the expected mass for an RNA oligo consisting of terminal 2',3' cyclic phosphate group.

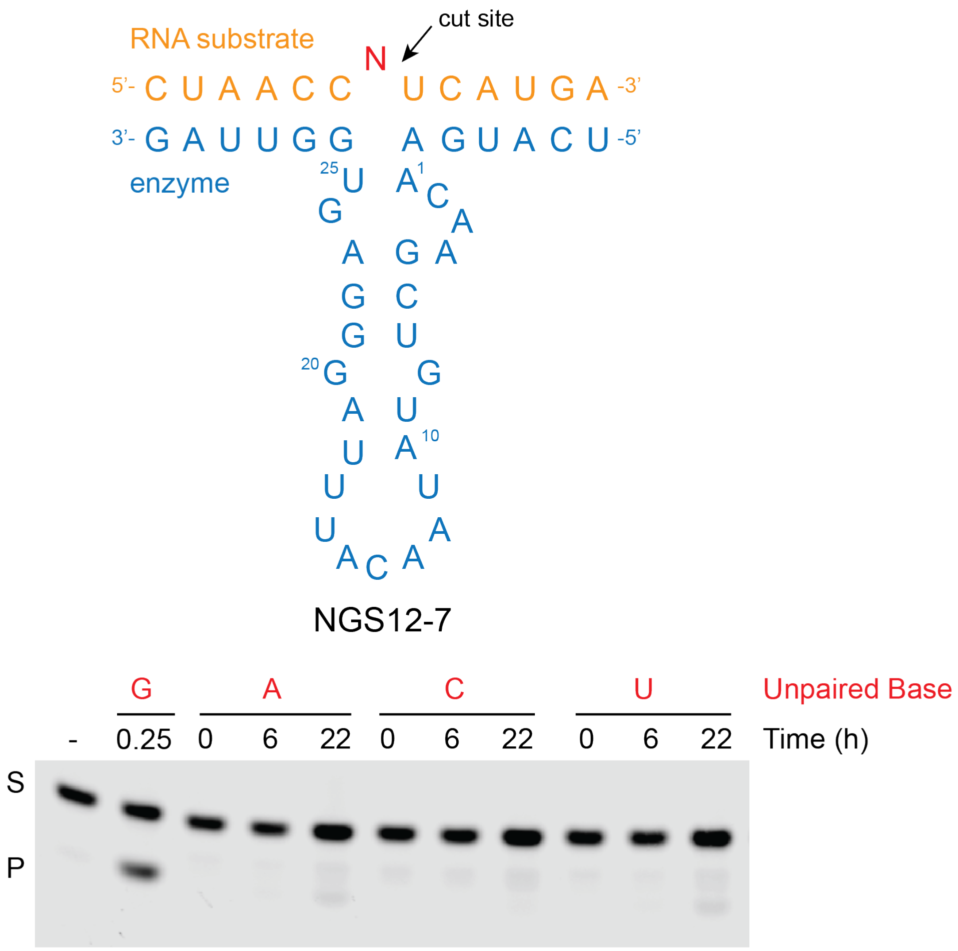

Supplementary Figure 8. **Nucleoside preference at the cleavage site**. Substitution of the unpaired ribo-G nucleotide by any of the other three nucleosides (A, C, or U) leads to enzyme inhibition. All reactions were performed in trans in 50 mM CHES (pH 8.5) containing 200 mM NaCl and 25 mM $MgCl_2$ at 23 °C under single-turnover conditions ([Fz] = 2.5 μM, [S] = 0.5 μM). S: substrate; P: cleavage product.

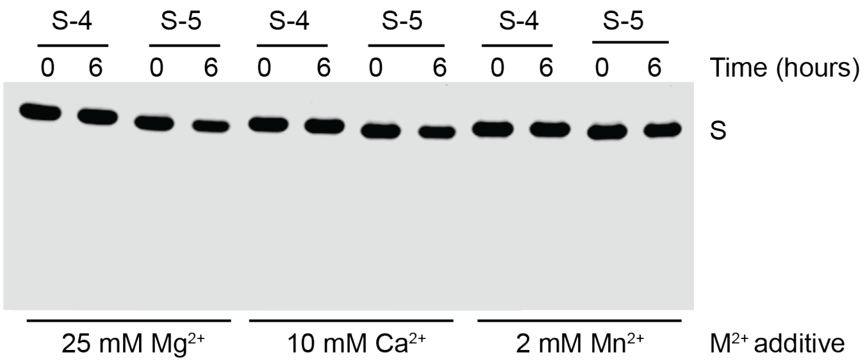

Supplementary Figure 9. **Cleavage of substrate S-4 and S-5 in the absence of NGS12-7**. Gel image showing the cleavage of S-4 and S-5 in the absence of NGS12-7. Reactions were performed in buffer (pH 8.5) containing 0.5 μM of substrate, 200 mM NaCl, supplemented with the labeled bivalent metal ion at 24 °C. S: substrate.

Left Panel

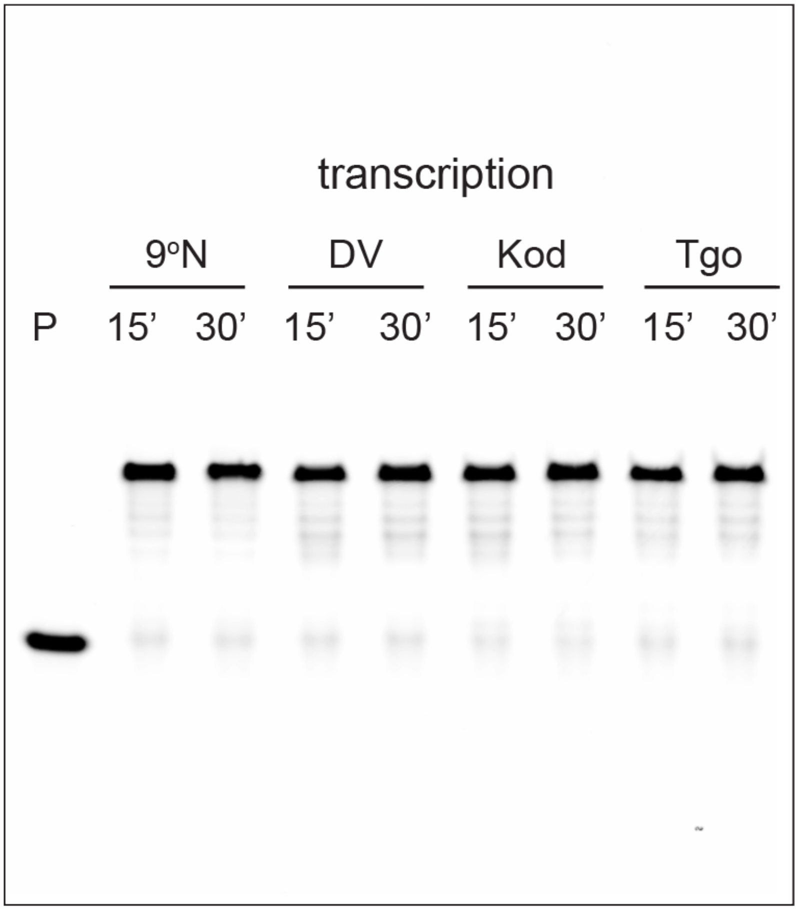

Right Panel

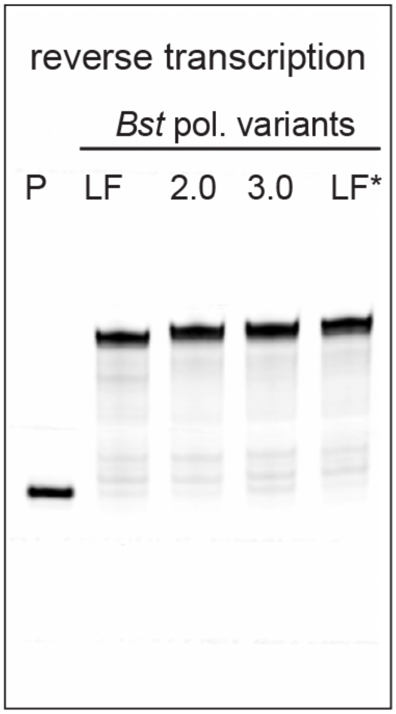

Supplementary Figure 10. Uncropped gel images for Figure 1. FANA transcription activity for wild-type archaeal DNA polymerases (exo$^-$) from 9°N, DV, Kod, and Tgo (left panel). Samples were analyzed after 15 and 30 minutes at 55°C. FANA reverse transcriptase activity of Bst DNA polymerase LF, 2.0, 3.0, and LF* (right panel). LF* denotes wild-type Bst DNA polymerase, large fragment, expressed and purified from *E. coli*. Samples were analyzed after 30 minutes at 50°C. All samples were resolved on denaturing PAGE and visualized using a LI-COR Odyssey CLx.

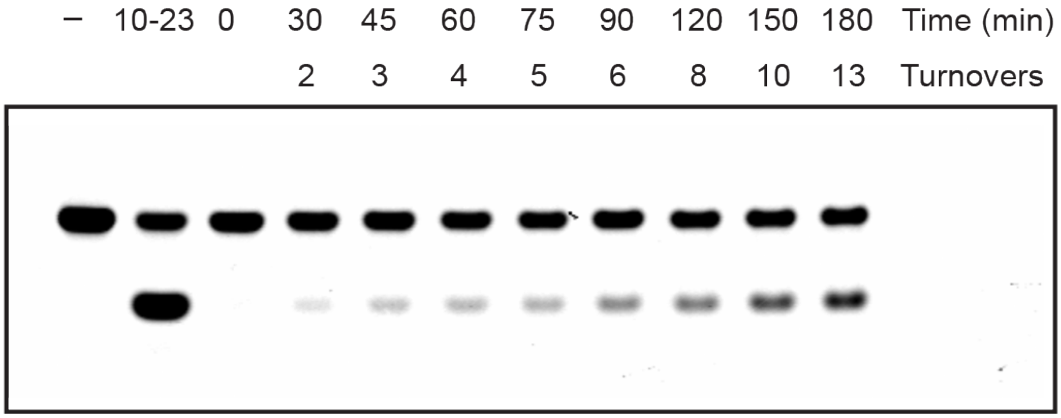

Supplementary Figure 11. Uncropped gel image for Figure 3e. Representative gel of NGS12-7 showing RNA cleavage activity under multiple-turnover conditions at pH 8.5, 24°C ([Fz] = 10 nM, [S] = 0.5 μM).

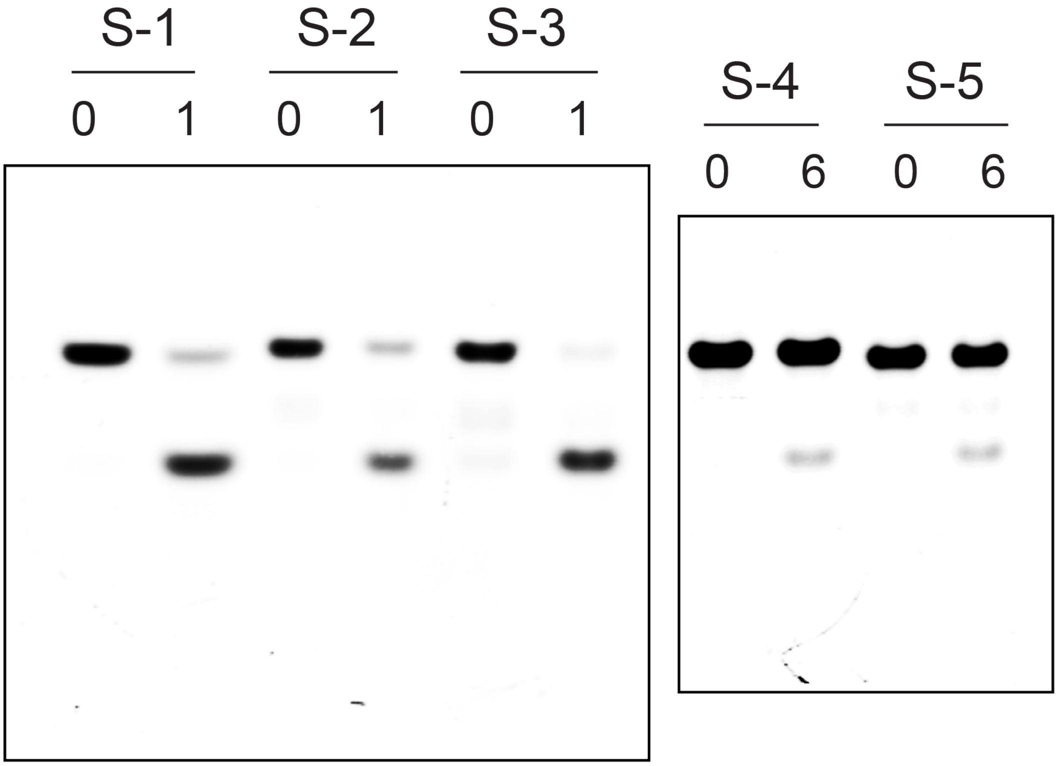

Supplementary Figure 12. Uncropped gel image for magnesium catalyzed reaction (row 1) in Figure 4f. Gel images showing the cleavage activity of engineered NGS12-7 variants toward different RNA substrates. Reactions were performed in buffer (pH 8.5) containing 200 mM NaCl supplemented with 25 mM magnesium chloride at 24 °C (Fz) = 2.5 μM, [S] = 0.5 μM). S: substrate; P: product.

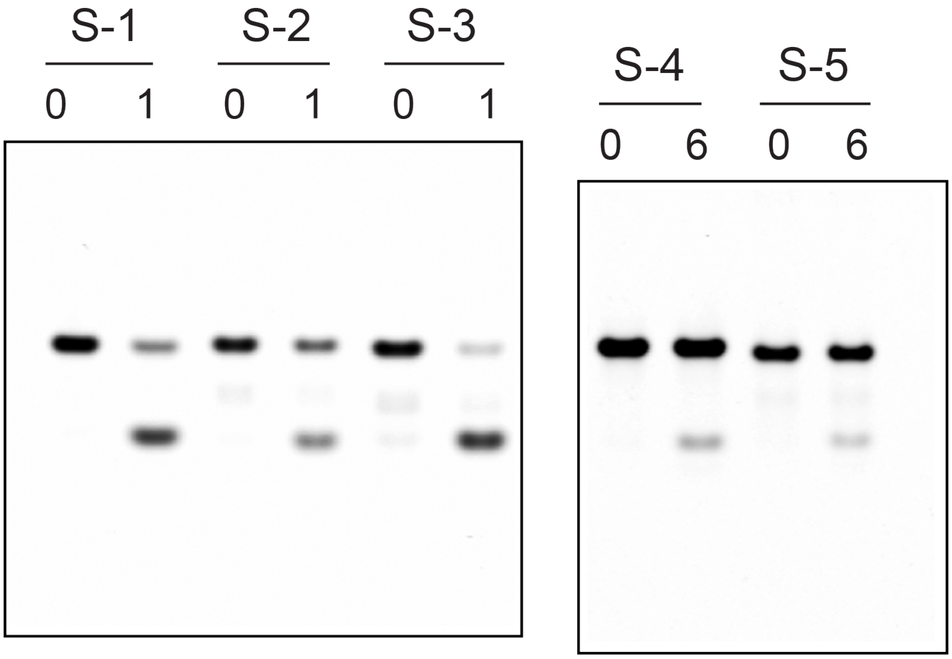

Supplementary Figure 13. Uncropped gel image for calcium catalyzed reaction (row 2) in Figure 4f. Gel images showing the cleavage activity of engineered NGS12-7 variants toward different RNA substrates. Reactions were performed in buffer (pH 8.5) containing 200 mM NaCl supplemented with 10 mM calcium chloride at 24 °C (Fz) = 2.5 μM, [S] = 0.5 μM). S: substrate; P: product.

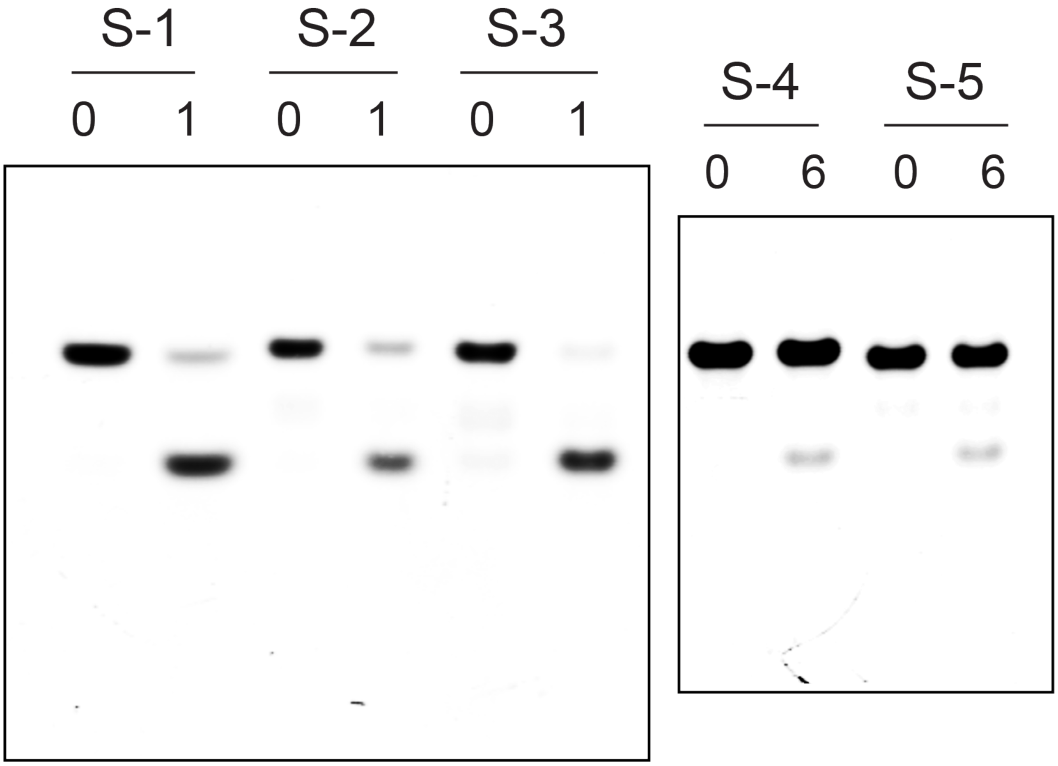

Supplementary Figure 14. Uncropped gel image for manganese catalyzed reaction (row 3) in Figure 4f. Gel images showing the cleavage activity of engineered NGS12-7 variants toward different RNA substrates. Reactions were performed in buffer (pH 8.5) containing 200 mM NaCl supplemented with 2 mM manganese chloride at 24 °C (Fz) = 2.5 μM, [S] = 0.5 μM). S: substrate; P: product.

Panel b

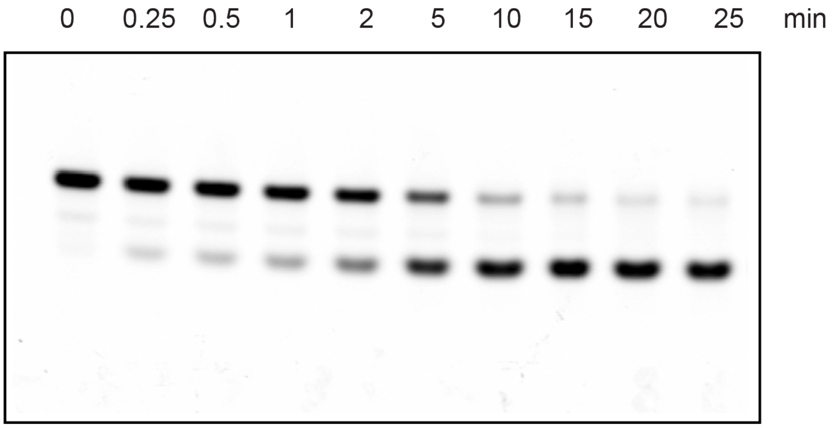

Panel c

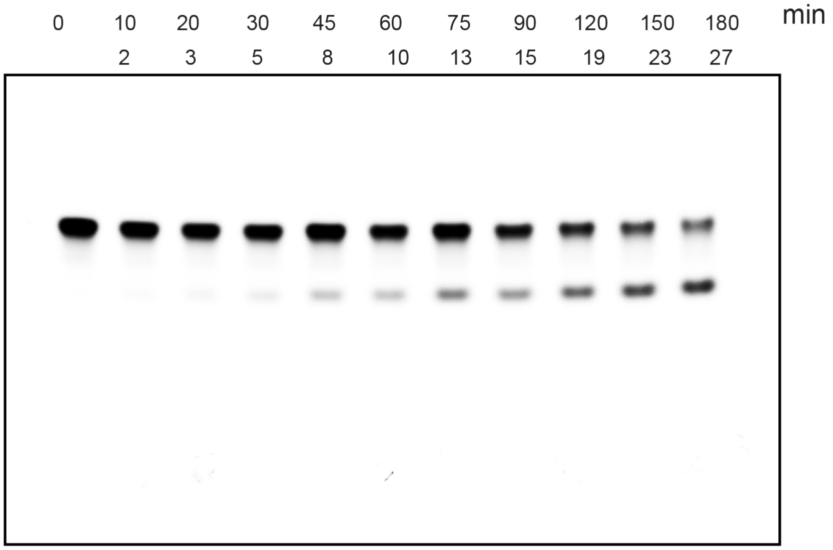

Supplementary Figure 15. Uncropped gel images for figure 5. Panel b. Substrate cleavage profile under single-turnover conditions. **Panel c**. Substrate cleavage under multiple-turnover conditions. ([Fz] = 10 nM, [S] = 0.5 μM). All reactions were carried out in trans in 50 mM CHES (pH 8.5) containing 200 mM NaCl and 25 mM $MgCl_2$ at 24 °C. S: substrate; P: cleavage product.

Supplementary Table 1. Complete table of oligonucleotide sequences used in this study.

Oligo Name	Sequence (5'-3')
Oligonucleotides for FANA replication studies	
30mer DNA	TCTCTATAGTGAGTCGTATAGGTGGTATCC
30mer FANA	ucucuauagugagucguauaggugguaucc
IR800-PBS8-short	/5IRD800/GGATACCACC
IR800-PBS9	/5IRD800/CTTTTAAGAACCGGACGAAC
IR800-PEG-PBS9	/5IRD800/AACAAACAAACAAACAAACAAACAAACAAACAAACAAACA /iSp18/CTTTTAAGAACCGGACGAAC
L16 library	GGATCGTCAGTGCATTGAGAATTCCTN_{40}(N=A:T:C:G=35:35:15:15)GTTC GTCCGGTTCTTAAAAG
PBS7	GGATCGTCAGTGCATTGAGA
PEG-PBS7	AACAAACAAACAAACAAACAAACAAACAAACAAACAAACA/iSp18/GGAT CGTCAGTGCATTGAGA
4NT9G	GGATCGTCAGTGCATTGAGATTAAGACTCGCCATGTTACGATCTGCCA AGTACAGCCTTGAATCGTCACTGGTGGTATCCCCTTGGGGA/3ddC
PBS8_extra	/56-FAM/CTTTTAAGAACCGGACGAACGTCCCCATGGGGATACCACC
Extra primer	CTTTTAAGAACCGGACGAAC
30mer HP	TCTCTATAGTGAGTCGTATAGGTGGTATCCGAAAGGATACCACC
Oligonucleotides for in vitro selection of FANA enzymes	
Biotin-fragment I	/5Biosg/AACAACAACAACAACAACAACAACAAAAAACCC
AllRNA-fragment II	/5Phos/GGGAAAAArCrCrUrArArCrCrGrUrCrArUrGrArC/iSp9//iFluorTG CGTCGGCCTACGCCAACAG
Splint	TTTTTCCCGGGTTTTT
L17 library	GGATCGTCAGTGCATTGAGACCTAACCN_{25}(N=A:T:C:G=25:25:25:25)TCA TGACCTGTTGGCGTAGGCCGACGC
PBS11	GCGTCGGCCTACGCCAACAG
PEG-PBS11	AACAAACAAACAAACAAACAAACAAACAAACAAACAAACA/iSp18/GCGT CGGCCTACGCCAACAG
RNA-blocker	GTCATGACGGTTAGG
Oligonucleotides for functional screening of sequences identified by Sanger and NGS sequencing	
RNA substrate	/5Cy5/rCrUrArArCrCrGrUrCrArUrGrA
16mer PBS11	CGGCCTACGCCAACAG
DNA template for ss12-1	CCTAACCACTCCCTAAATGTTACACAGCTTGTTCATGACCTGTTGGCGT AGGCCGACGCAACAACAACAACAACAACAAC
DNA template for ss12-3	CCTAACCACTCCCTAAATGTTACACAGCTTGTTCATGACCTGTTGGCGT AGGCCGACGCAACAACAACAACAACAACAAC
DNA template for ss12-11	CCTAACCAGAATGGCTAGTCAATGAAGCCCTATCATGACCTGTTGGCG TAGGCCGACGCAACAACAACAACAACAACAAC
DNA template for ss12-14	CCTAACCTAGGCCACTGAACGACCGACATGCATCATGACCTGTTGGCG TAGGCCGACGCAACAACAACAACAACAACAAC
DNA template for ss12-18	CCTAACCTCCAGGGTTGACCGGCATGAGGGCATCATGACCTGTTGGC GTAGGCCGACGCAACAACAACAACAACAACAAC
DNA template for ss12-19	CCTAACCACGAGTAGACGGGTCTGCCAGCTGCTCATGACCTGTTGGC GTAGGCCGACGCAACAACAACAACAACAACAAC
DNA template for ss12-20	CCTAACCTAGGCCACCGAGCGACCGACATGCATCATGACCTGTTGGC GTAGGCCGACGCAACAACAACAACAACAACAAC
DNA template for ss12-33	CCTAACCTCTCCCCTGCTGTACTAGAGGAAGTCATGACCTGTTGGCGT AGGCCGACGCAACAACAACAACAACAACAAC

DNA template for ss12-34	CCTAACCGCTGTGAGATCTACAGCACGGCACATCATGACCTGTTGGCGTAGGCCGACGCAACAACAACAACAACAACAAC
DNA template for ss12-36	CCTAACCGGATCTGATCATGCGCTGTGATATCATGACCTGTTGGCGTAGGCCGACGCAACAACAACAACAACAACAAC
DNA template for ss12-38	CCTAACCAACGAGGGATACATGAGGAAGATCATCATGACCTGTTGGCGTAGGCCGACGCAACAACAACAACAACAACAAC
DNA template for NGS12-1	CCTAACCACTCCCTAAATGTTACACAGCTTGTTCATGACCTGTTGGCGTAGGCCGACGCAACAACAACAACAACAACAAC
DNA template for NGS12-2	CCTAACCTAGGCTACTGAACGACCGACATGCATCATGACCTGTTGGCGTAGGCCGACGCAACAACAACAACAACAACAAC
DNA template for NGS12-3	CCTAACCTCAGCAGCGATCCGACACGTGGCCATCATGACCTGTTGGCGTAGGCCGACGCAACAACAACAACAACAACAAC
DNA template for NGS12-4	CCTAACCTCAACGGAGATCCGACAGCGCTGGCTCATGACCTGTTGGCGTAGGCCGACGCAACAACAACAACAACAACAAC
DNA template for NGS12-5	CCTAACCTCTGCAGCGATCCGACACGTGGCCATCATGACCTGTTGGCGTAGGCCGACGCAACAACAACAACAACAACAAC
DNA template for NGS12-6	CCTAACCCTAGGAGCATGGACGACCGACAGCATCATGACCTGTTGGCGTAGGCCGACGCAACAACAACAACAACAACAAC
DNA template for NGS12-7	CCTAACCACTCCCTAAATGTTATACAGCTTGTTCATGACCTGTTGGCGTAGGCCGACGCAACAACAACAACAACAACAAC
Oligonucleotides for structure and activity study	
DNA template for NGS12-8	CCTAACCACTCCCTAAATGATACACAGCTTGTTCATGACCTGTTGGCGTAGGCCGACGCAACAACAACAACAACAACAAC
DNA template for NGS12-10	CCTAACCACTCCCTAAATGTTGCACAGCTTGTTCATGACCTGTTGGCGTAGGCCGACGCAACAACAACAACAACAACAAC
DNA template for NGS12-11	CCTAACCACCCCCTAAATGTTACACAGCTTGTTCATGACCTGTTGGCGTAGGCCGACGCAACAACAACAACAACAACAAC
DNA template for NGS12-13	CCTAACCACTCCCTAAATGTTACGCAGCTTGTTCATGACCTGTTGGCGTAGGCCGACGCAACAACAACAACAACAACAAC
DNA template for NGS12-15	CCTAACCTCTCCCTAAATGTTACACAGCTTGTTCATGACCTGTTGGCGTAGGCCGACGCAACAACAACAACAACAACAAC
DNA template for NGS12-16	CCTAACCACTCCCTAAATGCTACACAGCTTGTTCATGACCTGTTGGCGTAGGCCGACGCAACAACAACAACAACAACAAC
DNA template for NGS12-18	CCTAACCGCTCCCTAAATGTTACACAGCTTGTTCATGACCTGTTGGCGTAGGCCGACGCAACAACAACAACAACAACAAC
DNA template for NGS12-19	CCTAACCACTCCCTAAATGTTACACAGCCTGTTCATGACCTGTTGGCGTAGGCCGACGCAACAACAACAACAACAACAAC
DNA template for NGS12-20	CCTAACCACTCCCTAAATGTTACACGGCTTGTTCATGACCTGTTGGCGTAGGCCGACGCAACAACAACAACAACAACAAC
Oligonucleotides for substrate specificity	
DNA template for NGS12-7 V2	CTTAACCACTCCCTAAATGTTATACAGCTTGTTCATGGCCTGTTGGCGTAGGCCGACGCAACAACAACAACAACAACAAC
DNA template for NGS12-7 V3	CTCAACCACTCCCTAAATGTTATACAGCTTGTTCATAGCCTGTTGGCGTAGGCCGACGCAACAACAACAACAACAACAAC
DNA template for NGS12-7 V4	GAGAGAGACTCCCTAAATGTTATACAGCTTGTTGGGTGCCTGTTGGCGTAGGCCGACGCAACAACAACAACAACAACAAC
DNA template for NGS12-7 V5	GGATTGGACTCCCTAAATGTTATACAGCTTGTTGTACTGCTGTTGGCGTAGGCCGACGCAACAACAACAACAACAACAAC
RNA substrate 2 (S2)	/5Cy5/rUrUrArArCrCrGrUrCrArUrGrG

RNA substrate 3 (S3)	/5Cy5/rUrCrArArCrCrGrUrCrArUrArG
RNA substrate 4 (S4)	/5Cy5/rGrArGrArGrArGrGrUrGrGrGrUrGrC
RNA substrate 5 (S5)	/5Cy5/rGrGrArUrUrGrGrGrUrGrUrArCrUrG
RNA substrate-rA	/5Cy5/rCrUrArArCrCrArUrCrArUrGrA
RNA substrate-rC	/5Cy5/rCrUrArArCrCrCrUrCrArUrGrA
RNA substrate-rU	/5Cy5/rCrUrArArCrCrUrUrCrArUrGrA
RNA substrate for cleavage product characterization	
RNA substrate with T_{24} DNA tail	/56-FAM/rCrUrArArCrCrGrUrCrArUrGrATTTTTTTTTTTTTTTTTTTTTTTT
DNA/RNA chimeric substrate	
Chimeric DNA-rG substrate	/5Cy5/CUAACCrGUCAUGA

Letters in lower cases denote FANA nucleotides; RNA residues are denoted by “r”; “N” in L16 and L17 libraries represents randomized positions; the underlined Ts in 4NT9G and PBS8_extra represent the T:T mismatch designed for fidelity measurement.

Supplementary Table 2. FANA sequences identified by Sanger sequencing

clone	reads	FANA sequence (catalytic core, 5'-3')	[a]trans-cleavage
ss12-1	5/25	ACAAGCUGUGUAACAUUUAGGGAGU	+++
ss12-3	1/25	UGCUUGUCGGCCGUCCAGUAGCCUA	-
ss12-11	1/25	UAGGGCUUCAUUGACUAGCCAUUCU	-
ss12-14	1/25	UGCAUGUCGGUCGUUCAGUGGCCUA	-
ss12-18	1/25	UGCCCUCAUGCCGGUCAACCCUGGA	-
ss12-19	1/25	GCAGCUGGCAGACCCGUCUACUCGU	-
ss12-20	1/25	UGCAUGUCGGUCGCUCGGUGGCCUA	-
ss12-33	1/25	CUUCCUCUAGUACAGCAGGGGAGA	-
ss12-34	1/25	UGUGCCGUGCUGUAGAUCUCACAGC	-
ss12-36	1/25	UAUCACAGCGCAUGAUCAGAUCC	-
ss12-38	1/25	UGAUCUUCCUCAUGUAUCCCUCGUU	-

[a] Reactions were performed in 50 mM Tris-HCl (pH 7.5) containing 200 mM NaCl and 10 mM $MgCl_2$ at 23 °C under single-turnover conditions ([Fz] = 2.5 μM, [S] = 0.5 μM). At least four time points were taken over a reaction time of 22 h.

Supplementary Table 3. Functional analysis of the most abundant NGS sequences

clone	[a]reads	FANA sequence (catalytic core, 5'-3')	reads in Sanger	[b]trans-cleavage
NGS12-1	1094604	ACAAGCUGUGUAACAUUUAGGGAGU	5/25	+++
NGS12-2	246993	UGCAUGUCGGUCGUUCAGUAGCCUA	0/25	-
NGS12-3	45914	UGGCCACGUGUCGGAUCGCUGCUGA	0/25	-
NGS12-4	42050	GCCAGCGCUGUCGGAUCUCCGUUGA	0/25	-
NGS12-5	31259	UGGCCACGUGUCGGAUCGCUGCAGA	1/25	-
NGS12-6	22563	UGCUGUCGGUCGUCCAUGCUCCUAG	0/25	-
NGS12-7	16471	ACAAGCUGUAUAACAUUUAGGGAGU	0/25	+++

[a]NGS analysis was performed on 8,000,000 processed reads from round 12. [b]Reactions were performed in 50 mM Tris-HCl (pH 7.5) containing 200 mM NaCl and 10 mM $MgCl_2$ at 23 °C under single-turnover conditions ([Fz] = 2.5 μM, [S] = 0.5 μM). At least four time points were taken over a reaction time of 22 h.

Supplementary Table 4. Highly abundant NGS sequences with homology to NGS12-1

clone	reads	FANA sequence (catalytic core, 5'-3')
NGS-1	1094604	A C A A G C U G U G U A A C A U U U A G G G A G U
NGS-7	16471	· · · · · · · · · A · · · · · · · · · · · · · · ·
NGS-8	15412	· · · · · · · · · · · · U · · · · · · · · · · · ·
NGS-10	13504	· · · · · · · · · · C · · · · · · · · · · · · · ·
NGS-11	12448	· G · ·
NGS-13	11525	· · · · · · · · C · · · · · · · · · · · · · · · ·
NGS-15	11006	· A
NGS-16	10914	· · · · · · · · · · · · G · · · · · · · · · · · ·
NGS-18	9967	· C
NGS-19	9448	· · · G ·
NGS-20	8224	· · · · · · C · · · · · · · · · · · · · · · · · ·

References

1. Nikoomanzar, A., Dunn, M. R. & Chaput, J. C. Engineered Polymerases with Altered Substrate Specificity: Expression and Purification. *Curr Protoc Nucleic Acid Chem* **69,** 4.75.1–4.75.20 (2017).
2. Nikoomanzar, A., Dunn, M. R. & Chaput, J. C. Evaluating the Rate and Substrate Specificity of Laboratory Evolved XNA Polymerases. *Anal. Chem.* **89,** 12622–12625 (2017).
